# Electron & Biomass Dynamics of *Cyanothece* Under Interacting Nitrogen & Carbon Limitations

**DOI:** 10.3389/fmicb.2021.617802

**Published:** 2021-04-09

**Authors:** Sophie Rabouille, Douglas A. Campbell, Takako Masuda, Tomáš Zavřel, Gábor Bernát, Lubos Polerecky, Kimberly Halsey, Meri Eichner, Eva Kotabová, Susanne Stephan, Martin Lukeš, Pascal Claquin, José Bonomi-Barufi, Ana Teresa Lombardi, Jan Červený, David J. Suggett, Mario Giordano, Jacco C. Kromkamp, Ondřej Prášil

**Affiliations:** ^1^Sorbonne Université, CNRS, LOV, Villefranche-sur-Mer, France; ^2^Sorbonne Université, CNRS, LOMIC, Banyuls-sur-Mer, France; ^3^Centre Algatech, Institute of Microbiology of the Czech Academy of Sciences, Třeboň, Czechia; ^4^Mount Allison University, Sackville, NB, Canada; ^5^Department of Adaptive Biotechnologies, Global Change Research Institute CAS, Brno, Czechia; ^6^Centre for Ecological Research, Balaton Limnological Institute, Klebelsberg Kuno u. 3. 8237 Tihany, Hungary; ^7^Department of Earth Sciences, Utrecht University, Utrecht, Netherlands; ^8^Department of Microbiology, Oregon State University, Corvallis, OR, United States; ^9^Max Planck Institute for Marine Microbiology, Bremen, Germany; ^10^Leibniz-Institute of Freshwater Ecology and Inland Fisheries, Zur alten Fischerhütte 2, Stechlin, Germany; ^11^Department of Ecology, Berlin Institute of Technology (TU Berlin), Ernst-Reuter-Platz 1, Berlin, Germany; ^12^UMR BOREA (CNRS 8067), MNHN, IRD (207), Université de Caen Basse-Normandie, Caen, France; ^13^Departamento de Botânica, Centro de Ciências Biológicas, Universidade Federal de Santa Catarina, Florianópolis, Brazil; ^14^Universidade Federal de São Carlos, São Carlos, Brazil; ^15^University of Technology Sydney, Climate Change Cluster, Faculty of Science, Ultimo, NSW, Australia; ^16^Dipartimento di Scienze della Vita e dell’Ambiente, UniversitaÌ Politecnica delle Marche, Ancona, Italy; ^17^NIOZ Royal Netherlands Institute for Sea Research and Utrecht University, Utrecht, Netherlands

**Keywords:** *Cyanothece*, *Crocosphaera subtropica*, photosynthesis, light limitation, carbon limitation, nitrogen fixation

## Abstract

Marine diazotrophs are a diverse group with key roles in biogeochemical fluxes linked to primary productivity. The unicellular, diazotrophic cyanobacterium *Cyanothece* is widely found in coastal, subtropical oceans. We analyze the consequences of diazotrophy on growth efficiency, compared to NO_3_^–^-supported growth in *Cyanothece*, to understand how cells cope with N_2_-fixation when they also have to face carbon limitation, which may transiently affect populations in coastal environments or during blooms of phytoplankton communities. When grown in obligate diazotrophy, cells face the double burden of a more ATP-demanding N-acquisition mode and additional metabolic losses imposed by the transient storage of reducing potential as carbohydrate, compared to a hypothetical N_2_ assimilation directly driven by photosynthetic electron transport. Further, this energetic burden imposed by N_2_-fixation could not be alleviated, despite the high irradiance level within the cultures, because photosynthesis was limited by the availability of dissolved inorganic carbon (DIC), and possibly by a constrained capacity for carbon storage. DIC limitation exacerbates the costs on growth imposed by nitrogen fixation. Therefore, the competitive efficiency of diazotrophs could be hindered in areas with insufficient renewal of dissolved gases and/or with intense phytoplankton biomass that both decrease available light energy and draw the DIC level down.

## Introduction

While the structures and genetic regulation of the key enzymes of photosynthesis and nitrogen (N_2_) fixation are relatively well understood, we lack understanding of how these two processes interact, in particular under fluctuating environmental conditions. Regulation of N_2_-fixation in photosynthetic diazotrophs is especially important, as molecular oxygen (O_2_), a product of photosynthesis, irreversibly denatures nitrogenase (Gallon, 1992), the key enzyme responsible for N_2_-fixation. Autotrophic, unicellular cyanobacterial diazotrophs within the known groups B and C (UCYN-B, C; Zehr et al., 2001; Taniuchi et al., 2012) overcome nitrogenase inhibition by O_2_ through temporal separation of day-time photosynthesis from N_2_-fixation (Fay, 1992; Gallon, 1992; Bergman et al., 1997) fueled by night-time respiration of carbon reserves accumulated during previous day-time photosynthesis. Despite this temporal offset, the yield of N_2_-fixation remains tightly dependent upon the efficiency of photosynthesis (Agawin et al., 2007; Großkopf and LaRoche, 2012; Grimaud et al., 2014). Further, the ability of UCYN to meet their nitrogen requirements using either N_2_ or NO_3_^–^ provides them with more flexibility in fluctuating environments. For instance, Agawin et al. (2007) demonstrated that a unicellular diazotroph can efficiently compete against nitrogen-limited phytoplankton incapable of N_2_-fixation, if sufficient light energy is available to support the extra costs of diazotrophy. We expect the respective demands and allocations of photosynthetic reductant in cells grown on nitrate or under obligate diazotrophy to differ because of the stoichiometries of these N assimilation pathways, but also because of their different timing in the light cycle, which imply distinct metabolic routes. It is therefore still unclear how reductant and energy demands are affected by the source of nitrogen and how much more costly it really is, for a diazotroph to grow on N_2_ *vs* NO_3_^–^.

Because UCYN so much rely on dynamic internal reserves of carbon, an efficient photosynthesis and storage is key to their success. We therefore raise the question of the regulation of cellular processes by dissolved inorganic carbon (DIC) availability. As discussed by Stoll et al. (2019), DIC limitation occurred over glacial cycles which periodically triggered upregulations of the algal carbon concentrating mechanism (CCM). CCM is an essential component of the photosynthetic machinery as it maintains the intracellular CO_2_ concentration (Badger et al., 1998; Badger et al., 2006; Xu et al., 2008; Rae et al., 2013), enhancing the growth efficiency. Although absolute DIC limitation is rare in the open ocean, Riebesell et al. (1993) argued that depending on which inorganic carbon forms can be used under non-limiting light and nutrient conditions, the growth rate of diatoms could actually be limited by the supply of CO_2_. Such observation, made from a microbial primary producer, may hint towards similar effects in diazotrophic cyanobacteria. Should DIC become transiently limiting, it could affect both the immediate photosynthetic efficiency and the subsequent activity of N_2_-fixation in UCYN. In coastal environments and freshwater systems, where DIC concentrations are lower than in the open ocean, phytoplankton blooms may draw down DIC to levels where the absolute amount of DIC can become limiting for growth, at least transiently. So far, the literature demonstrates that DIC limitation can be a main determinant of phytoplankton growth in estuaries (Fogel et al., 1992) as well as in brackish waters during red tide episodes (Hansen et al., 2007). The development of harmful algal blooms leads to conditions of DIC limitation, in which cyanobacteria are likely to outcompete eukaryotic phytoplankton (O’Neil et al., 2012; Van Dam et al., 2018) and we therefore wonder whether such conditions could also constitute temporary niches for diazotrophs. Not only Lu et al. (2019) reported the presence of N_2_-fixing, cyanobacterial strains during a cyanobacteria-dominated harmful algal bloom (HAB) but they also demonstrated and their facilitating role on the onset of the toxic bloom. The photosynthetic efficiency in intertidal communities of diatom-dominated microphytobenthos is also periodically constrained by DIC availability (Vieira et al., 2016; Marques da Silva et al., 2017). In freshwater systems, Kragh and Sand-Jensen (2018) challenged the paradigm that nutrients or light limit primary production in lakes, showing that DIC is a strong limiting factor, especially in the soft-water lakes. DIC limitation, or coupled P and DIC limitation, was also found in Saint Anna Lake in Transylvania (Lajos Vörös, personal communication). DIC limitation also controls primary production in alkaline ponds (Zeng et al., 2019).

Thus this work addresses two questions. First, the consequences of diazotrophy on growth efficiency, compared to NO_3_^–^-supported growth, are not yet quantified. Second, we wonder how UCYN cope with the energetic burden of N_2_-fixation should they also have to face carbon limitation. Selected strains in the genus *Cyanothece* have extensively been studied as model organisms to address various physiological or gene regulation features (Schneegurt et al., 1994; Cólon-López et al., 1997; Sherman et al., 1998; Toepel et al., 2008; Bandyopadhyay et al., 2013). *Cyanothece* is also becoming an emerging model for studying the performance of diazotrophy during the natural diel cycles of photosynthesis and N_2_-fixation in coastal environments (Rabouille et al., 2014; Aryal et al., 2018, Sicora et al., 2019), where it naturally occurs. The issue of a possible DIC limitation is therefore all the more relevant for this genus. We chose the strain *Cyanothece* sp. ATCC 51142 (hereafter *Cyanothece*), recently re-classified as *Crocosphaera subtropic*a ATCC 51142 (Mareš et al., 2019)). As a starting point, we consider the theoretical energy and reducing power requirements of photosynthesis, N_2_-fixation and NO_3_^–^ uptake, to compare the direct costs of these two N assimilatory pathways. Double arrows indicate a multiple step conversion. N_2_-fixation and NO_3_^–^ acquisition are distinct growth modes that impose different reductant and ATP burdens upon cells, as illustrated by the stoichiometric reactions involved (Falkowski and Raven, 2007):

Photosynthetic Electron Transport:

(1) 2HO2+8H++out4hν→∼12H++inO+24e-

(2) 14H++in3ADP+3P→i14H++out3ATP

Carbon Fixation:

(3) CO+2external1ATP→CO+2internal1ADP+Pi

(4) CO+2internal4e+-3ATP→CHO2+3ADP+3Pi

where CO_2__external_ refers to the extracellular CO_2_ and CO_2__internal_ to the intracellular CO_2_. Note that in this study we do not distinguish between the form(s) of inorganic carbon used by cells and abbreviate them as CO_2_.

Nitrogen Assimilation:

(5) NO+3-8e+-1ATP→→NHif4+NOis3-theNsource

(6) 1/2N+24e+-8ATP→→NH4++1/2H+28ADP+8P⁢ifi⁢N⁢is2⁢the⁢N⁢source

$${\mathrm{NH}}_{4}^{+}+\alpha -\mathrm{ketoglutarate}+\mathrm{ATP}+2\,e^{-}\to \to$$

(7) $$\mathrm{glutamate}\,\left(\mathrm{organic}\,N\right)+\mathrm{ADP}+P_{i}$$

Carbohydrate Re-oxidation:

(8) $${\mathrm{CH}}_{2}O\to \to {\mathrm{CO}}_{2}+4e^{-}$$

Aerobic Respiration:

(9) $$4\,e^{-}+O_{2}+6\,A\,D\,P+6\,P_{i}\to 2\,H_{2}\,O+6ATP$$

Linear photosynthetic electron transport (eqn. 1) generates ∼ 4 e^–^ and translocates ∼ 12 H^+^ across the thylakoid membranes per two water molecules oxidized at Photosystem II (PSII). Concurrently, 3 ATP molecules are synthesized during one full rotation of the AtpC subunit of the ATP synthase, driven by 14 H^+^ flowing through the membrane. Hence, linear electron transport generates 3 ATP × 12/14 ≈ 2.6 ATP per two water molecules oxidized. Combining eqn. 1 and 2 to express this ratio in terms of electrons per ATP yields 1.55 e^–^/ATP. In contrast, the reductant/ATP ratio needed for the Calvin cycle is 2NADPH/3ATP = 1.33 e^–^/ATP. Achieving sufficient reductant to ATP ratio, depending on the demands of cellular processes, is, at least partly, tuned by the cyclic electron transport around PSI generating only ATP (for further details, see e.g. Bernát and Rögner, 2011; Kramer and Evans, 2011). Carbon assimilation into biomass then costs 4 e^–^ and 4 ATP per carbon atom (eqn. 3 & 4), including a nominal cost of 1 ATP/CO_2__*external*_ to account for the cost of CCM moving CO_2__*external*_ to CO_2__*internal*_ (Raven et al., 2014). Every N atom assimilated from NO_3_^–^ to organic N (glutamate) costs 1 ATP per NO_3_^–^ for uptake (Flores et al., 2005), followed by 8 + 2 = 10 e^–^ and one ATP for reductive assimilation (eqn. 5 & 7). In contrast, N assimilated from N_2_ costs 4 + 2 = 6 e^–^ but 8 + 1 = 9 ATP (eqn. 6 & 7). To the extent the H_2_ byproduct of N_2_-fixation is re-captured by dehydrogenase activity (Tamagnini et al., 2007; Wilson et al., 2012), the net metabolic reductant cost further drops towards 4 e^–^ per N for diazotrophy, whilst further protecting the nitrogenase against oxygen toxicity (Zhang et al., 2014).

Thus, in a cell with a C:N ratio of 7:1, growing on NO_3_^–^, the direct photosynthetic generation of a C7:N1 biomass implies an allocation to C assimilation of ∼7 × 4 = 28 e^–^ and ∼7 × 4 = 28 ATP and an allocation of 10 e^–^ and 2 ATP to N assimilation. This represents an investment of 38 e^–^ and 30 ATP overall per C7:N1. The same cell growing with N_2_ as N source with a hypothetical direct photosynthetic generation of a C7:N1 biomass would make the same ∼28 e^–^ and ∼28 ATP allocation to C assimilation, but 4–6 e^–^ and 9 ATP towards N assimilation, i.e. 32–34 e^–^ and 37 ATP total.

Thus, there is a lower electrons demand but higher ATP demand for photosynthetic growth under diazotrophy. Alternately stated, when growing on NO_3_^–^ the photosynthetic production of biomass uses ∼38 e^–^/30 ATP ≈ 1.27 e^–^/ATP ratio, closely matching the output of the photosynthetic electron transport coupled to proton translocation, i.e. 1.33 e^–^/ATP. In contrast, photosynthetic diazotrophy requires only a 32–34 e^–^/37 ATP ≈ 0.9 e^–^/ATP ratio to accumulate the same biomass, resulting in a mismatch between the output of (linear) photosynthetic electron transport and metabolic requirements. Hence, these alternative modes of growth could cause differences in photosynthetic resource allocation and performance. Note that these estimates shift further if NO_3_^–^ or N_2_ assimilation are fueled indirectly by respiratory oxidation of carbohydrate because the yield of ATP/e^–^ then depends upon the relative allocation of electrons to respiratory electron transport *vs*. recycling into assimilatory paths. Furthermore, the assembly, protection, and daily *de novo* (re)synthesis of the iron-rich, labile nitrogenase complex imposes additional costs upon diazotrophic cells (Großkopf and LaRoche, 2012), which are expected to be higher than the cost of maintenance of the more stable enzymes of the NO_3_^–^/NO_2_^–^ uptake and reductase system.

While NO_3_^–^ assimilation can occur in the light and, therefore, be directly provisioned with electrons and ATP by photosynthesis, *Cyanothece* fixes N_2_ during the night, using both electrons and ATP generated at the expense of respiration of previously stored carbon reserves. Thus, each electron and ATP equivalent ultimately destined for N_2_-fixation passes transiently through a CH_2_O reserve stage, increasing the instantaneous burden upon the carbon assimilation system during the photoperiod. Efficient carbon acquisition and storage is therefore essential for the diazotrophic growth of *Cyanothece*. It is, however, unknown how the processes of photosynthesis, carbon storage and N_2_-fixation interact to optimize light saturated growth with dissolved inorganic carbon (DIC) that transitions from replete to a possible DIC limitation during the day, and back to DIC replete conditions during the night. These temporal shifts in DIC availability further exacerbate the competition for electrons between CO_2_ and nitrogen sources.

In the following, we assess the energetic demands of *Cyanothece* under diazotrophic growth versus reductive assimilation of NO_3_^–^ and we describe the related electron transport and biomass dynamics when a periodical DIC limitation negatively affects carbon fixation. Cultures were continuously maintained in a state of exponential growth using bioreactors run in turbidostat mode. Using these systems, we simultaneously monitored photosynthetic electron transport, the electron requirement for carbon fixation, as well as carbon and nitrogen acquisition and incorporation in cells. We also evaluated whether the additional energy demand for N_2_-fixation translated into greater carbon storage or consumption. All these parameters were obtained from a variety of analyses, some of which are described in more details in the Supplementary material.

## Materials and Methods

**FIGURE 1:**
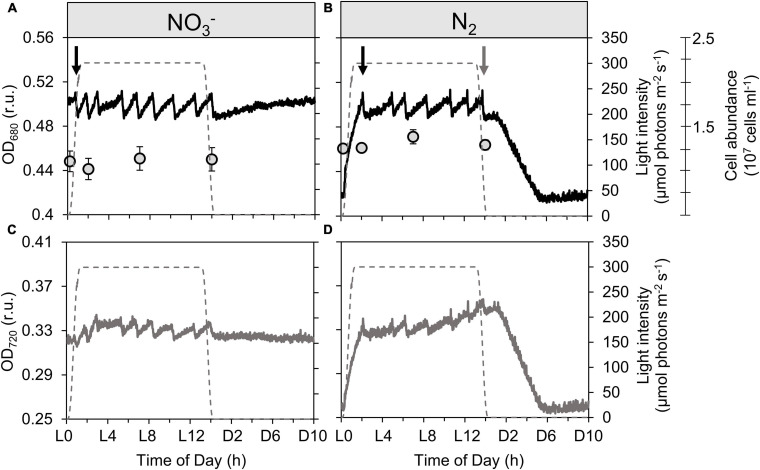
Record of dynamics in optical density measured at 680 nm [black lines; OD_680_, **(A,B)**] and 720 nm [gray lines; OD_720_, **(C,D)**] together with cell abundance [gray circles; **(A,B)**] of *Cyanothece* turbidostat cultures cultivated in ASP 2 medium supplemented with NO_3_^–^ **(A,C)** and under obligate diazotrophy **(B,D)**. The OD_680_ (more sensitive to changes in Chl *a* content) and OD_720_ (more sensitive to changes in non-pigment biomass) signals are measured within the photobioreactor vessel and represent a typical record of optical density over a diel cycle. The cell abundance data are averages from 9 samples from 3 independent bioreactors for each treatment; error bars show standard deviations. The gray dashed lines show the light profile throughout the day. Black arrows point to the first culture dilution event at the beginning of each light phase, while the gray arrow points to the last dilution event in the N_2_-fixing culture.

**FIGURE 2:**
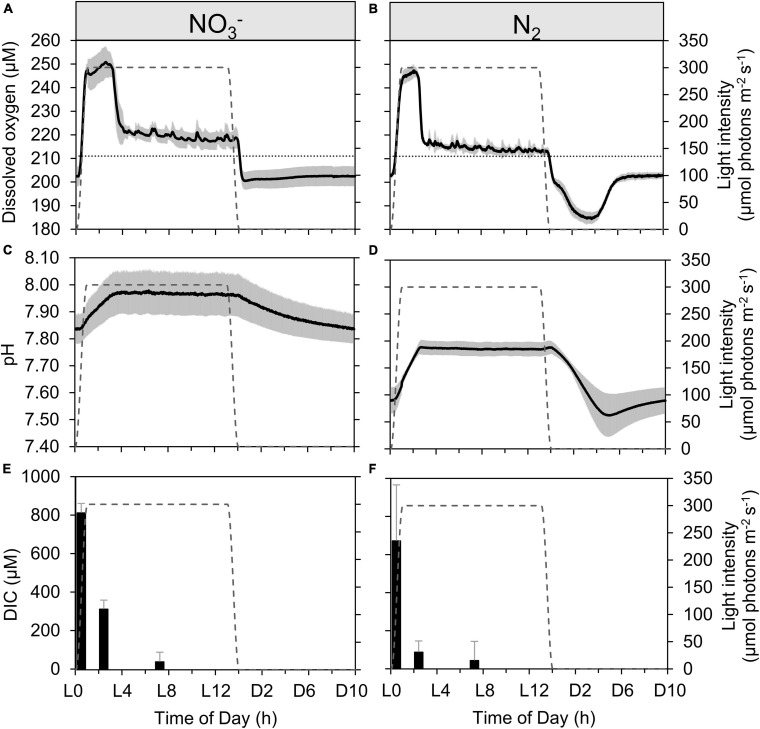
Dynamics of dissolved oxygen **(A,B)**, pH **(C,D)**, and dissolved inorganic carbon [DIC, **(E,F)**] in continuous *Cyanothece* cultures grown in ASP 2 medium either supplemented with NO_3_^–^ **(A,C,E)** or not **(B,D,F)**, throughout the daily 14h/10h light/dark cycle. Each trace represents averages of 5–6 light/dark cycles of 2 **(A–D)** or 3 **(E)** (with the exception of L3 sample with *n* = 2), **(F)** independent cultures, with error intervals and bars, respectively (representing standard deviations). The dotted lines in panels **(A,B)** represent the level of O_2_ equilibrium at 28°C in the medium, while the gray dashed line in each panel represents the light profile throughout the day.

The core of this study uses a close monitoring of the dynamics of the photosynthetic apparatus (detailed below) to trace electron fluxes through/around photosystems, with parallel assessments of the related carbon fluxes, carbon and nitrogen storage, and of the overall growth efficiency. The monitoring was completed with a series of bulk analyses (cell counts, biomass, cellular C and N contents, and an assessment of carbon allocation using Fourier Transform Infrared Spectroscopy (FTIR, Supplementary Figure 1)). UV-Vis spectroscopy was applied to determine the pigment composition of the cells including Chl *a*, carotenoids and phycobiliproteins (Table 1), while Photosystem I to Photosystem II (PSI:PSII) and phycobilisomes to PSII abundance ratios were derived using low temperature (77K) fluorescence emission spectroscopy (Supplementary Figure 2). The specific growth rates were derived from changes in optical density (Supplementary Figure 3, Figure 1). Online monitoring of dissolved O_2_ concentrations in the cultures informed on the net production or consumption of oxygen (Figure 2 and Supplementary Figure 4). Last, photosynthetic efficiency was monitored using a membrane inlet mass spectrometer (MIMS). Specifically, gross and net O_2_ fluxes in light and dark were measured using an ^18^O_2_-based approach that allows for differentiating between photosynthetic O_2_ evolution and light-dependent O_2_ uptake by Mehler reaction and photorespiration (Supplementary Figure 5). Additionally, CO_2_ draw down by the cultures was monitored while simultaneously observing O_2_ fluxes (Supplementary Figure 6; see Supplementary material). We detail below the conditions under which the experiments were performed as well as the methodology related to the photosynthetic activity measurements, which constitute the core of the work. The methods related to all other parameters can be found in the Supplementary material.

**TABLE 1:** Morphology and composition of *Cyanothece* cells cultivated in ASP2 medium supplemented with nitrate (NO_3_^–^) or under obligate diazotrophic (N_2_) conditions, normalized per cell.

Parameter	Unit	*Cyanothece* nitrate (NO_3_^–^) cultures							*Cyanothece* diazotrophic (N_2_) cultures						
		L0	L2	L7	L14 = D0	D4	Light phase average	Daily average	L0	L2	L7	L14 = D0	D4	Light phase average	Daily average

		Onset of light phase	DIC replete light phase	DIC limited light phase	DIC limited light phase	Dark phase			Onset of light phase	DIC replete light phase	DIC limited light phase	DIC limited light phase	Dark phase		
Specific growth rate	(h^–1^)	0.067 ± 0.012	0.064 ± 0.024	0.030 ± 0.020	0.015 ± 0.005	−	0.026 ± 0.018	0.013 ± 0.021	0.164 ± 0.025	0.082 ± 0.027	0.017 ± 0.036	0.013 ± 0.005	−	0.034 ± 0.040	0.009 ± 0.050
Doubling time	(h)	10.4 ± 1.9	10.8 ± 4.1	23.0 ± 15.3	46.7 ± 14.5	−	26.5 ± 17.8	53.2 ± 86.4	4.2 ± 0.6	8.5 ± 2.8	40.9 ± 87.7	53.8 ± 22.9	−	20.1 ± 23.2	79.2 ± 448.5
Cellular diameter	(μm)	3.03 ± 0.09	3.15 ± 0.08	3.08 ± 0.08	3.11 ± 0.11	−	3.09 ± 0.09	−	2.92 ± 0.07	3.02 ± 0.08	2.97 ± 0.07	2.93 ± 0.04	−	2.96 ± 0.07	−
Cellular dry weight	(fg dry weight cell^–1^)	6378 ± 368	−	7854	8542	−	7591 ± 1106	−	5716 ± 650	8000	−	7907 ± 1203	−	7208 ± 1293	−
Chlorophyll *a* (chl *a*)	(fg chl *a* cell^–1^)	144 ± 9	154 ± 15	132 ± 8	143 ± 17	−	143 ± 14	−	109 ± 14	112 ± 18	106 ± 22	102 ± 19	−	107 ± 18	−
Carotenoids	(fg Σcarotenoids cell^–1^)	40 ± 3	45 ± 5	42 ± 4	44 ± 5	−	43 ± 5	−	39 ± 5	40 ± 6	40 ± 7	39 ± 5	−	40 ± 5	−
Phycocyanin (PC)	(fg PC cell^–1^)	479 ± 183	476 ± 152	449 ± 133	505 ± 83	−	477 ± 20	−	358 ± 41	423± 38	334 73	390 ± 22	−	376 ± 33	−
Allophycocyanin (APC)	(fg APC cell^–1^)	213 ± 117	267 ± 120	236 ± 101	228 ± 48	−	236 ± 20	−	165 ± 26	198 ± 21	163 ± 43	183 ± 12	−	177 ± 14	−
Phycobilisomes	(fg Σ (PC+APC) cell^–1^)	692 ± 463	743 ± 410	685 ± 357	733 ± 195	−	713 ± 25	−	522 ± 102	621 ± 86	497 ± 170	573 ± 49	−	553 ± 48	−
Polysaccharides (PS)	(fg glucose eq. PS cell^–1^)	1978 ± 976	2620 ± 801	2831 ± 713	3081 ± 1038	−	2593 ± 920	−	1657 ± 702	3083 ± 566	3899 ± 982	3766 ± 838	−	3197 ± 1141	−
Cyanophycin (CP)	(fg arginine eq. CP cell^–1^)	−	77 ± 26	115 ± 19	51 ± 32	75 ± 16	81 ± 39	−	−	62 ± 11	26 ± 4	23 ± 1	47 ± 6	37 ± 20	−
Cellular carbon	(fg C cell^–1^)	2515 ± 159	−	2973	3297	−	2928 ± 393	−	237 ± 523	3219	−	3213 ± 291	−	2936 ± 485	−
Cellular nitrogen	(fg N cell^–1^)	584 ± 30	−	668	741	−	664 ± 78	−	534 ± 69	586	−	510 ± 27	−	543 ± 39	−
C:N ratio	w:w	4.30 ± 0.35	−	4.45	4.45	−	4.40 ± 0.09	−	4.45 ± 0.58	5.50	−	6.30 ± 0.66	−	5.42 ± 0.93	−
C:N ratio	mol:mol	5.02 ± 0.08	−	5.20	5.19	−	5.14 ± 0.10	−	5.19 ± 0.08	6.41	−	7.35 ± 0.49	−	6.31 ± 1.03	−

*The four time points represent the onset of light phase (L0), DIC replete light phase (L2) and DIC limited light phase (L7, L14) (see Figure 1). Data represent averages from samples taken over successive days in 3 culture replicates, error intervals represent standard deviations. When no error intervals are available, *n* ≤ 2.*

### Cyanobacteria Culturing

*Cyanothece* sp. ATCC 51142 was cultivated in flat panel FMT-150 photobioreactors (Photon Systems Instruments, Drásov, Czech Republic) of 400 mL or 1000 mL volume at 28°C in ASP 2 medium (Provasoli et al., 1957; van Baalen, 1962) buffered with 15 mM TAPS (pH of the medium was set to 7.8 by addition of a few drops of 1 M NaOH). Since the pioneering works of Reddy et al. (1993), ASP 2 medium has been widely used to grow the genus *Cyanothece*. ASP 2 medium was either prepared without addition of inorganic nitrogen (for N_2_ fixing cultures, thereafter called N_2_-fixing cultures.) or supplemented with NO_3_^–^ (17.6 mM) for the nitrate-rich cultures (thereafter called NO_3_^–^ cultures). Both these treatments were run in triplicate and all cultures were bubbled with ambient air (∼410 ppm CO_2_) with a flow rate of 200 mL min^−1^, as controlled by a gas monitoring system described in detail in (Červený et al., 2009). The air was supplied to the culture through U-shape metal tubes with four perforations of 0.7 mm diameter along the bottom part. This resulted in an average bubble size of diameter 2.6 mm (Nedbal et al., 2010). A vent topped with an air filter (Minisart, hydrophobic, 0.2 μm) connected the culture headspace with the atmosphere. Additional cultures were run with the exact same setup but with a regulated CO_2_ supply as a control for DIC replete cultures.

Illumination was provided by cool-white LEDs following 14h:10h L:D daily cycles with maximal light intensity set to 300 μmol photons m^–2^ s^–1^. The light intensity of the initial and the final hour of each light cycle was set to follow a sinusoidal increase and decrease, respectively. Built in probes mounted within the reactors allowed a real-time, continuous monitoring of dissolved O_2_ (InPro6800 electrode), pH and temperature (InPro3253, both probes are manufactured by Mettler-Toledo Inc, Columbus, OH, USA), the yield of steady-state chlorophyll *a* (Chl *a*) fluorescence emission using an built in pulse-amplitude modulated system, as well as optical density measured at as well as OD_720_ (as a 428 proxy for light scattering) and OD_680_ (as a proxy for both 429 light scattering and Chl *a* concentration). The pH and dO_2_ probes were calibrated before and after the experiment while the OD sensors were calibrated only before the experiments, both according to the instructions of the manufacturer. Cultures were run in turbidostat mode controlled by the OD_680_ signal (see the Supplementary file for detailed methodology). The specific growth rate in each culture was determined by exponential fitting of the OD_720_ signal (Supplementary Figure 3) provided by the built in sensor during the turbidostat mode, according to (Zavřel et al., 2015).

*Cyanothece* was cultivated continuously for at least 21 days, and sampled regularly four times a day after cultures had reached equilibrium. Time is expressed in hours into the 14h:10h light cycle: the prefix L indicates hours in the light (from L0 to L14) and the prefix D means hours into the dark (from D0 to D10). Samples were taken at the onset of the light (L0), after two hours of light (L2), at mid-light phase (i.e. after 7h of light, L7), and at the time of light to dark transition (L14 = D0). Some additional samples were also taken after 1h of light (L1) and after 2 or 4 hours of darkness (D2 and D4, respectively).

### Photosynthetic Activity Measurements

#### PSII Kinetics

Variable fluorescence data were collected using a FastOcean Fast Repetition Rate fluorimeter (FRRf, Chelsea Technologies Group, West Molesey, Surrey, UK) on culture samples acclimated to low light (ca. 5 μmol photons m^–2^ s^–1^ for at least 20 min) to ensure full oxidation of the electron transport chain prior to measurements, as described previously (Suggett et al., 2015). Briefly, the FRRf was set to deliver a single turnover induction protocol of 100 flashlets over ca. 200 μs (2 μs flashlet pitch). Excitation was provided from a bank of blue and orange LEDs (setting of E_*LED*_ 450 nm of 1.10 and E_*LED*_ 624 nm of 1.26) to ensure full and consistent Q_*A*_ reduction. Measurements were recorded as an average of 10 consecutive acquisitions applied at intervals of 200 ms. Each FRRf acquisition was then fitted to the KPF model (Kolber et al., 1998) using FastPRO8 software (Chelsea Technologies Group) to yield the minimum (F_0_, or F′) and maximum (F_*M*,_ or F_*M*_′) PSII fluorescence, PSII absorption cross-section (σ_*PSII*,_ or σ_*PSII*_′; nm^2^ photon^–1^) for dark and actinic light (′) conditions. After the single turnover induction to close PSII the flashlet pitch was slowed to allow estimation of the lifetime for re-opening of PSII by downstream electron transport (τ or τ′, μs). All fluorescence yields were adjusted for baseline fluorescence retrieved from parallel FRRf measurements upon filtrates of each sample passed through a 0.22 μm pore filter (cellulose acetate, syringe filter; Whatman, USA). Values of σ_*PSII*_ were taxonomically weighted to the composite blue-orange excitation spectra and thus spectrally adjusted to match a white spectrum (Suggett et al., 2004). We used these parameters to quantify PSII electron transport during the light cycle, following:

(10) ePSII-s-1=-1σ/PSII(FV/FM)×Y×III(photonsms-2)-1

where Y_*II*_ is the effective quantum yield of photosystem II photochemistry, calculated as

(11) Y=II(F-M′F′)/FM′

#### PSI Kinetics

PSI parameters were determined using a Dual-PAM-100 measuring system (Walz GmbH, Effeltrich, Germany). 3 mL of cell suspension were filtered through a GF/F filter and placed between the perspex rods of the emitter (DUAL-E) and detector (DUAL-DR) units of the system using a DUAL-B leaf holder. A default “induction with recovery” protocol was run after 2 min of dark adaptation, with 10 s of initial far red illumination followed by 40 s darkness and, subsequently, by 200 μmol photons m^–2^ s^–1^ red actinic light with saturating pulses (30 ms, 10.000 μmol photons m^–2^ s^–1^) on top. These saturating pulses (SPs) served to probe maximal P_700_ levels in the dark and during the actinic illumination (Pm and Pm′, respectively). SPs were given at 20 s intervals over the actinic light period and the average of signal intensities at pulses 2 to 5 (4 pulses) were considered in order to minimize the effect of state transitions on the PSI quantum yields [Y_(I)_] that might have been induced by the dark adaptation. Raw data were corrected by subtracting a blank value of about 19.5 mV, determined by using a wet filter paper without *Cyanothece* cells. ETR(I) was subsequently normalized according to Pm values (to take into account changes in PSI abundance) and Chl *a* concentration. Rates of P_700_ re-reduction kinetics were calculated from manually determined t_1__/__2_ values using saturating pulses during the initial dark period of the “induction with recovery” protocol.

#### NAD(P)H Kinetics

Light-induced formation of NAD(P)H as well as its post-illumination reduction to NAD(P)^+^, and concomitant changes in Chl *a* fluorescence was probed using cell suspensions filled into a standard 1 cm quartz cuvette placed in the Optical Unit ED-101US/MD of the Dual-PAM-100 measuring system (Walz). Simultaneous detection of NAD(P)H and Chl *a* fluorescence was performed using a NADPH/9-AA module (consisting DUAL-ENADPH and DUAL-DNADPH) combined with DUAL-DPD and DUAL-DR (Schreiber and Klughammer, 2009). Before measurements, samples were spun down and resuspended in fresh ASP 2 medium supplemented with NO_3_^–^ (nitrate-rich cultures) or not (obligate diazotrophic cultures), for a final Chl *a* concentration of 7 μmol–1 followed by 10 min of dark adaptation. Then, 60 s of 200 μmol photons m^–2^ s^–1^ red actinic light was applied to initiate photosynthetic electron transport and NAD(P)H and Chl *a* fluorescence were simultaneously recorded. Dark NAD(P)H reduction level was estimated by dividing the signal increase upon illumination with the total signal amplitude between the maximum upon illumination [NAD(P)H] and post-illumination minimum [NAD(P)^+^]:

(12) 100×[NAD(P)H]/([NAD(P)H]+[NAD(P)]+)

Post-illumination increase of the Chl *a* fluorescence (if any) was interpreted as reduction of the PQ pool (Deng et al., 2003).

## Results and Discussion

Continuous *Cyanothece* cultures were cultivated in photobioreactors in two distinct growth regimes: either (i) in standard ASP 2 medium supplemented with NO_3_^–^ as nitrogen source, or (ii) in a modified ASP 2 medium without NO_3_^–^, to impose dependence upon N_2_-fixation. Overall, and across our 4-day monitoring during the equilibrium phase (within a 19-day experiment; see Materials & Methods), the average growth rates are 0.22 ± 0.07 d^–1^ in N_2_-fixing cultures and 0.30 ± 0.08 d^–1^ in the NO_3_^–^ cultures. These values are significantly different (p < 0.05, ANOVA, Tukey HSD test, n = 7 consecutive days for N_2_-fixing cultures and 11 for NO_3_^–^ cultures) and reflect the overall lower growth in cells growing as obligate diazotrophs compared to cells growing on NO_3_^–^. When integrated from the transient, hourly growth phases (Tables 1, 2 and Supplementary Figure 3), the daily mean and SD values of the specific growth rates are 0.21 ± 1.19 d^–1^ in the N_2_-fixing cultures and 0.31 ± 0.51 d^–1^ in the NO_3_^–^ cultures. The large standard deviations illustrate that instantaneous, metabolic processes are highly dynamic and lead to significant variability in transient cell growth processes within a 24 h period. For instance, and as discussed below, apparent negative growth rates obtained in the early dark phase in the N_2_-fixing cultures are related to the significant consumption of carbon reserves to fuel N_2_-fixation, which led to a decrease in the overall carbon biomass (hence in optical density). *Cyanothece* has achieved much higher growth rates in other studies (see for instance Reddy et al., 1993; Agawin et al., 2007; Brauer et al., 2013), and we believe the lower achieved growth in our bioreactor experiments results from transient DIC limitations. In an ocean acidification scenario with elevated pCO_2_ and DIC levels (380 vs. 980 μatm pCO_2_, 1970 vs. 2150 μmol kg^–1^ DIC), increased particulate organic carbon (POC) and nitrogen (PON) production rates in *Cyanothece* were attributed to lowered energy costs for the CCM (Eichner et al., 2014). Similarly, *Crocosphaera* grown diazotrophically under low pCO_2_ treatment (180 ppm) shows a lowered growth rate compared to a high pCO_2_ (800 ppm), which was attributed to an ATP deficit due to the extra energy invested in the CCM (Garcia et al., 2013). As we will see below, the efficiency of CCM in the studied *Cyanothece* is limited and, in addition to the energy cost to operate these mechanisms, a remaining carbon limitation likely still hinders carbon acquisition in both treatments as DIC levels in the bioreactors are drawn down.

### Biomass Buildup and Transient Growth Dynamics Within the Light Cycle

As cell sizes differ slightly, but significantly, between the two treatments (mean comparison test, n = 32, p < 0.001), the concentration of all major cellular components are expressed on both cell (Table 1) and biovolume bases (Table 2). On average, diameters of cells grown on NO_3_^–^ are 5% larger (3.12 ± 0.05 μm vs. 2.96 ± 0.05 μm; Table 1) and accumulate 19% more nitrogen, in particular in pigments and nitrogen storage pools (see Supplementary Material). In contrast, although the daily average of total cellular carbon content is similar in both types of cultures, and they also both show a diurnal increase in cellular carbon content, the diel pattern of a carbon storage strategy is more pronounced in the N_2_-fixing cultures. FTIR spectra reveal striking differences in carbon allocation into carbon reserves and their temporal dynamics (Supplementary Figure 1) with, in particular, larger allocation into the carbohydrate pools and a storage buildup strategy in the N_2_-fixing cultures, while the NO_3_^–^ cultures tend to accumulate more proteins. The carbohydrate content of the cells determined by FTIR increases linearly during the light period. By the end of the light period, the carbohydrate content of the NO_3_^–^ culture increased by 50%, while carbohydrate content of the N_2_-fixing culture increased by 230%, compared to onset of light (Supplementary Figure 1). The strategy of *Cyanothece* to store significant carbon reserves in the light to operate a respiratory protection of nitrogenase and to fuel N_2_-fixation during the dark is well known (Reddy et al., 1993; Schneegurt et al., 1994). We further see here that this strategy is quite adaptable as, when provided with NO_3_^–^, the extra energy that N_2_-fixation required is no longer needed and *Cyanothece* accumulates far lower reserves in the light.

**TABLE 2:** Morphology and composition of *Cyanothece* cells cultivated in ASP2 medium supplemented with nitrate (NO_3_^–^) or under obligate diazotrophic (N_2_) conditions, normalized per biovolume.

Parameter	Unit	*Cyanothece* nitrate (NO_3_^–^) cultures						*Cyanothece* diazotrophic (N_2_) cultures					
			
		L0	L2	L7	L14 = D0	Light phase average	Daily average	L0	L2	L7	L14 = D0	Light phase average	Daily average

		Onset of light phase	DIC replete light phase	DIC limited light phase	DIC limited light phase			Onset of light phase	DIC replete light phase	DIC limited light phase	DIC limited light phase		
Specific growth rate	(h^–1^)	0.067 ± 0.012	0.064 ± 0.024	0.030 ± 0.020	0.015 ± 0.005	0.026 ± 0.018	0.013 ± 0.021	0.164 ± 0.025	0.082 ± 0.027	0.017 ± 0.036	0.013 ± 0.005	0.034 ± 0.040	0.009 ± 0.050
Doubling time	(h)	10.4 ± 1.9	10.8 ± 4.1	23.0 ± 15.3	46.7 ± 14.5	26.5 ± 17.8	53.2 ± 86.4	4.2 ± 0.6	8.5 ± 2.8	40.9 ± 87.7	53.8 ± 22.9	20.1 ± 23.2	79.2 ± 448.5
Cellular volume	(μm^3^)	14.66 ± 1.27	16.35 ± 1.27	15.33 ± 1.23	15.83 ± 1.67	15.52 ± 1.42	−	13.11 ± 0.91	14.47 ± 1.08	13.68 ± 0.98	13.14 ± 0.57	13.60 ± 1.03	−
Cellular dry weight	(fg μm^–3^)	415 ± 32	−	481	493	463 ± 42	−	428 ± 20	588	−	592 ± 81	536 ± 93	−
Chlorophyll *a*	(fg μm^–3^)	9.81 ± 0.36	9.39 ± 0.51	8.62 ± 0.47	9.06 ± 0.75	9.23 ± 0.67	−	8.30 ± 1.10	7.71 ± 1.17	7.70 ± 1.34	7.77 ± 1.24	7.87 ± 1.18	−
Carotenoids	(fg μm^–3^)	2.76 ± 0.13	2.75 ± 0.14	2.76 ± 0.12	2.79 ± 0.17	2.76 ± 0.13	−	2.99 ± 0.26	2.77 ± 0.32	2.89 ± 0.36	2.99 ± 0.28	2.91 ± 0.30	−
Phycocyanin	(fg μm^–3^)	32.7 ± 12.8	29.2 ± 9.6	29.3 ± 9.0	31.9 ± 6.2	30.4 ± 9.4	−	27.3 ± 3.6	29.2 ± 3.4	24.4 ± 5.6	29.7 ± 2.1	27.0 ± 3.7	−
Allophycocyanin	(fg μm^–3^)	14.5 ± 8.1	16.3 ± 7.5	15.4 ± 6.7	14.4 ± 3.4	15.4 ± 6.4	−	12.6 ± 2.2	13.7 ± 1.8	11.9 ± 3.2	13.9 ± 1.1	12.7 ± 2.1	−
Phycobilisomes	(fg μm^–3^)	47.2 ± 31.8	45.4 ± 25.3	44.7 ± 23.6	46.3 ± 13.3	45.8 ± 23.5	−	39.8 ± 8.2	42.9 ± 6.8	36.3 ± 12.7	43.6 ± 4.2	39.7 ± 8.0	−
Polysaccharides	(fg μm^–3^)	135 ± 62	159 ± 42	185 ± 44	191 ± 48	166 ± 52	−	130 ± 62	213 ± 36	290 ± 94	287 ± 65	237 ± 91	−
Cellular carbon	(fg μm^–3^)	164 ± 14	−	18 ± 2	19 ± 0	179 ± 14	−	174 ± 23	23 ± 7	−	240 ± 18	217 ± 37	−
Cellular nitrogen	(fg μm^–3^)	38.0 ± 2.7	−	40.9	42.7	40.5 ± 2.4	−	39.2 ± 2.2	43.0	−	38.2 ± 0.3	40.1 ± 2.6	−
C:N ratio		4.30 ± 0.47	−	4.45	4.45	4.40 ± 0.09	−	4.45 ± 0.64	5.50	−	6.30 ± 0.46	5.42 ± 0.93	−

*The four time points represent the onset of light phase (L0), DIC replete light phase (L2) and DIC limited light phase (L7, L14) (see Figure 1). Data represent averages from samples taken over successive days in 3 culture replicates, error intervals represent standard deviations. When no error intervals are available, *n* ≤ 2.*

Slight differences can be observed in the temporal dynamics of proteins, which can be related to the N acquisition strategy (nitrate uptake in the light phase *vs*. N_2_-fixation in the dark phase), as shown by the analysis of FTIR spectra (see Supplementary Material). However, the amplitude of protein fluctuations is quantitatively comparable in the two treatments and the protein content of both cultures is similar at the end of the light phase (Supplementary Figure 1).

In the subsections below, we use biochemical data to describe the growth dynamics observed in each treatment within the light cycle. We will see that cultures pass through two distinct phases during the light period: firstly, a DIC-replete, light-saturated phase that lasts for about 3 h in the N_2_-fixing cultures and 4h in the NO_3_^–^ cultures, followed by a DIC-limited phase for the rest of the light period. Then, clear differences in their behavior appear in the dark, related to N_2_-fixation in the N_2_-fixing cultures.

Changes in cell abundance and composition are visualized by monitoring OD_680_ and OD_720_. Figure 1 shows the high-frequency data obtained with the probes recording OD_680_ and OD_720_ in real-time within the cultures. Typically, in any given culture, OD rises as biomass increases; when cultures are run in turbidostat mode, dilution is controlled so as to maintain the biomass concentration close to the setpoint (in this case, an OD_680_ setpoint). Therefore, each time the set OD_680_ threshold is reached, the automated pump activates to replace the culture with new medium, thereby diluting the biomass concentration; this leads to a drop in the OD signal(s), which then acts as a stop signal for the pump. In the following, we are describing growth processes at shorter time scales than the cell division cycle, which result in transient changes in the cell C and N composition, thereby affecting the OD signal. These processes can for instance be carbon incorporation, carbon consumption through respiration, biomass buildup from reserves, nitrogen incorporation, etc. Therefore, the “growth” we describe using the OD proxy encompasses all of these processes. As we will see in section Growth Dynamics During the Dark Phase, OD_720_ is particularly sensitive to changes in the refractile intracellular POC. When growth processes are relatively constant, successive dilutions occur at rather regular time intervals in the cultures, as often as needed to maintain the culture density near the desired value, which lead to zigzagging trends in the OD records. As we will see below (see section Growth Dynamics During the Dark Phase), OD_720_ is particularly sensitive to changes in the refractile intracellular POC, which makes it a suitable proxy to describe the dynamics of the carbohydrate pools.

#### Growth Dynamics During the Light Phase

Unless something changes in the cultures conditions, the dilution events are expected to go on at regular intervals, for the entire light phase, as carbon is incorporated. The visible disruption in dilution events, as observed by the slower increase in density and so, the absence of any dilution event between ∼L3 and L5 in both NO_3_^–^ and N_2_-fixing cultures shows something changed in the overall physiology of cells from about L3—L4. Using these results and the concomitantly logged levels of dissolved oxygen and pH (Figure 2), we identified a series of physiological phases in the diel dynamics of *Cyanothece.* The dynamics of ODs compared to [O_2_] provides a deeper insight on this phenomenon. The OD_720_ signal was also used to derive the average, daily growth rate, as well as the transient rates of growth observed at the scale of hours (Supplementary Figure 3).

A very high, instantaneous growth rate (0.16 ± 0.02 h^–1^) is derived from the rapid increase in OD_720_ at the onset of the light period in the N_2_-fixing culture (Figure 1B and Supplementary Figure 3B), which reflects a buildup of C stores. Deschamps et al. (2008) identified these storage compounds as being polysaccharides. Conversely, the NO_3_^–^ culture shows only minor variability in OD (Figures 1A,C); the already high OD_720_ at the dark to light transition suggests that persisting cellular carbon reserves are rather significant, consequent to a lower carbon demand during dark hours in NO_3_^–^ cultures (Figure 1C). The instantaneous growth rate in NO_3_^–^ cultures in the first hours of light period thus appears much lower (0.06 ± 0.01 h^–1^; Supplementary Figure 3A) compared to the N_2_-fixing cultures. Over these early light hours, the level of dissolved oxygen increases rapidly in both types of cultures as a result of photosynthetic O_2_ evolution initiated by the increasing irradiance (from dark to 300 μmol photons m^–2^ s^–1^; Figures 2A,B). The pH in the N_2_-fixing cultures increases faster (0.095 h^–1^) compared to the NO_3_^–^ cultures (0.049 h^–1^; Figures 2C,D) due to a rapid DIC consumption, which decreases the DIC concentration to less than a third of that observed in the NO_3_^–^ cultures (Figures 2E,F). The pH decrease in the N_2_-fixing cultures during the dark phase was not compensated due to the weaker TAPS buffering capacity below pH 7.7.

After the initial rapid increase, and as irradiance stabilizes, the O_2_ concentration increases only slightly: 5.14 ± 0.05 μmol O_2_ L^–1^ h^–1^ in the NO_3_^–^ cultures and 2.41 ± 0.02 μmol O_2_ L^–1^ h^–1^ in the N_2_-fixing cultures (derived from the O_2_ signal as shown in Figures 2A,B). This stage corresponds to light-limited photosynthesis, at a rate sufficient to maintain O_2_ concentration significantly above the physical equilibrium expected from the bubbling. When grown under favorable conditions under a 12:12 light:dark regime, oxygen evolution in *Cyanothece* peaks around the mid-light phase to L8, following a progressive photoacclimation of PSII centers, before decreasing in the second half of the light phase (Meunier et al., 1997, 1998). In the present experiments, the increasing stage is interrupted well before mid-light as DIC becomes limiting at about L3.5 in the NO_3_^–^ cultures and L2.5 in the N_2_-fixing cultures (Figures 2E,F). The onset of DIC limitation coincides with a massive decline in photosynthetic O_2_ production in both cultures, as seen by the decrease in the concentration of dissolved O_2_ (Figures 2A,B). The O_2_ level starts to decline earlier in the N_2_-fixing cultures, in correlation with their much faster depletion of DIC (Figures 2E,F). As a consequence, instantaneous growth rates progressively revert to very low values (Supplementary Figure 3B). The decline in photosynthetic rates following the draw-down of DIC was confirmed by trends in ^13^C incorporation shown in a companion paper (Polerecky et al., 2021), with a decrease in C specific assimilation rates from 1.7 ± 0.35 d^–1^ (morning) to 0.39 ± d^–1^ (afternoon) in N_2_-fixing cultures and from 0.84 ± 0.15 d^–1^ (morning) to 0.42 ± 0.07 d^–1^ (afternoon) in NO_3_^–^ cultures (Polerecky et al., 2021). When CO_2_ concentration in the inflow air is increased, the DIC limitation is relaxed and O_2_ concentration increases during the light phase (Supplementary Figure 4). The dO_2_ dynamics under DIC limitation was indeed different from a setup where DIC limitation was prevented and where no sharp decline of photosynthetic activity during the day could be observed (Červený and Nedbal, 2009).

During the light period, electrons provided by photosynthetic water-splitting in the N_2_-fixing culture cells are allocated for CO_2_ fixation, while none are used directly for N acquisition. In contrast, in cells growing on NO_3_^–^ as a nitrogen source diurnal patterns in NO_3_^–^ assimilation follow those in C assimilation, with highest rates in both processes measured in the morning (Polerecky et al., 2021). NO_3_^–^ reduction requires 8 + 2 electrons per NO_3_^–^ ion assimilated to glutamate (see Introduction), originating ultimately from the photosynthetic electron transport. Hence, these cells must divert at least some of the electrons generated by photosynthesis from carbon fixation to NO_3_^–^ reduction. This concurrent and competing demand for photosynthetic electrons limits the rate of DIC drawdown in the NO_3_^–^ culture as compared to the N_2_-fixing culture that draws down the DIC pool sooner (Figure 2F). Therefore, light saturated photosynthesis can proceed a little longer into the light period in the NO_3_^–^ grown cells before the DIC pool becomes depleted (Figures 2A,B,E,F). This agrees with the theoretical, comparative electron demand based upon C:N ratios for each metabolic strategy outlined earlier, multiplied by their respective growth rates.

For the remaining 10 h of the light period, the O_2_ concentration decreases slowly at an average rate of −0.40 μmol O_2_ L^–1^ h^–1^ in the NO_3_^–^ cultures and −0.27 μmol O_2_ L^–1^ h^–1^ in the N_2_-fixing cultures (Figures 2A,B). Nonetheless, the oxygen concentration remains super-saturated above the equilibrium concentration, indicating a lower, but persistent photosynthetic activity in both types of cultures, which is sufficient to outrun the equilibration driven by bubbling with air. During this phase, O_2_ evolution approaches a compensation point among DIC-limited photosynthesis, respiration, and O_2_ exchange due to bubbling. MIMS data (see Supplementary Material) confirm the observed robust DIC drawdown and show that net O_2_ evolution is the same between treatments while respiratory and light-dependent O_2_ uptake is higher in the NO_3_^–^ culture (Supplementary Figure 5). Concomitantly, the successive increases in OD triggering dilution events confirm that growth recovers at least partially during this period of sustained DIC limitation (Figure 1) and both cultures show an equivalent growth rate of 0.02 ± 0.01 h^–1^. This recovery most probably reflects induction of CCM (Supplementary Figure 6) to counter the drawdown of DIC. DIC concentrations decreased to about 50 μmol L^–1^, irrespective of the growth regime, by the middle of the day (Figures 2E,F). Assuming a balanced carbonate system, at the pH of the cultures (pH of ∼8) the CO_2__*aq*_: DIC ratio is predicted to be ∼1:100, implying a minimum CO_2_ concentration of ca. 0.5 μmol L^–1^ CO_2_ in the bioreactors. This is 2–3 orders of magnitude lower than the typical *K*_*M*_ of cyanobacterial RuBisCO (105–185 μmol CO_2_ L^–1^; Badger et al., 1998), suggesting also that high activity of carbon concentrating mechanisms (CCM) are required to supply RubisCO.

We note that the lower pH in the N_2_-fixing cultures could result in additional costs for maintaining pH homeostasis and/or fueling N_2_-fixation (Shi et al., 2012; Luo et al., 2019). However, the pH difference between culture types of 0.02 ± 0.004 (Figure 2) was small enough to consider this effect negligible (< 5%), compared to the larger differences in the energetic costs of N_2_ fixation (equation 5) vs. NO_3_^–^ assimilation (equation 6).

#### Growth Dynamics During the Dark Phase

An immediate drop in O_2_ concentration is clearly visible at the onset of the dark period as soon as photosynthesis stops, which is likely accentuated by the upregulation of the terminal electron acceptor of the respiratory chain (the cytochrome c oxidase, Stöckel et al., 2008) and increased respiration upon the onset of the dark (Meunier et al., 1997). O_2_ concentrations decline to a sub-saturation level in both N_2_ and NO_3_^–^ cultures (dotted lines, Figures 2A,B), indicative of sustained respiratory activity that is not fully compensated by the continuous bubbling of the turbidostats. However, the distinct OD dynamics and O_2_ fluxes suggest that different processes operate under the two nitrogen regimes.

In the NO_3_^–^ culture, the O_2_ concentration decreases to 201 μmol L^–1^, stabilizes for almost half an hour and then slightly and monotonically increases throughout the dark period. Sub-saturated oxygen concentrations remain for the entire dark phase, indicative of a basal, dark respiration activity. The concomitant OD signal shows first an initial decrease in OD_680_ due to the last dilution event (Figure 1B). Then OD_680_ slowly increases while OD_720_ slowly decreases during the night (Figures 1A,C). We hypothesize that these dynamics are due to pigment synthesis (increase in OD_680_) and respiration of refractile carbohydrate storage granules resulting in a decrease in (OD_720__)._

**FIGURE 3:**
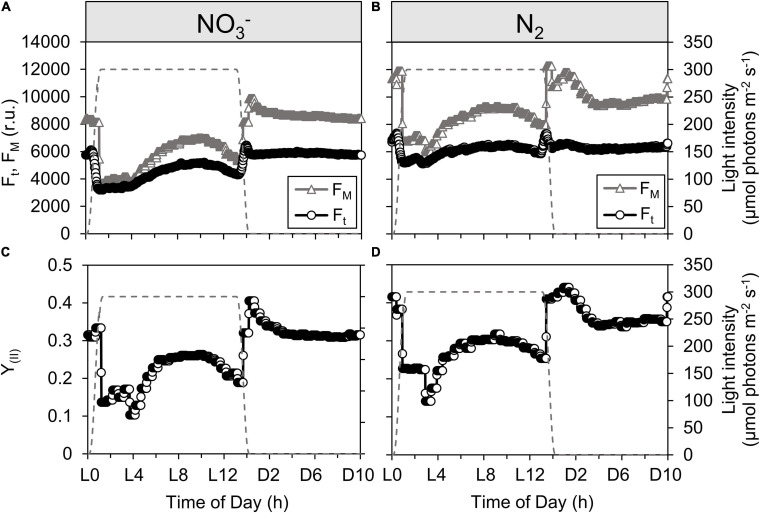
Dynamics of steady-state fluorescence yield, F_*t*_ [open circles; **(A,B)**], maximal fluorescence yields, F_*M*_ and F_*M*_ [open triangles; **(A,B)**] and quantum yield of PSII, Y*II* [open circles; **(C,D)**] during *Cyanothece* cultivation in ASP 2 medium supplemented with NO3.. **(A,C)** or under obligate diazotrophy **(B,D)**. The traces of F_*t*_, F*_M_* (or F*_M_′*) and Y_*II*_ represent one typical record throughout a 24 h period acquired by the online pulse-amplitude modulated sensors of the photobioreactors. The gray dashed line represents the light profile over a 24 h period.

In the N_2_-fixing culture, after an initial decrease in OD (caused by the last dilution event; Figure 1B, grey downward arrow), followed by a short (∼1 h) stabilization phase, both OD_680_ and OD_720_ decrease linearly for over 5 h. The overall decrease in OD matches that of the initial increase in the light period. Since neither cell divisions (León et al., 1986; Dron et al., 2013) nor dilution events occur in the dark, the rapidly decreasing OD signals suggests a decline in the refractile intracellular particulate material. Moreover, in contrast to the NO_3_^–^ culture with relatively stable O_2_ in the darkness, the O_2_ concentration in the N_2_-fixing culture shows a remarkable transient decrease (to 184 μmol L^–1^) over the initial 4 h of darkness (Figure 2B). This O_2_ dynamic exposes the oxygen demand associated with the respiratory requirements for N_2_-fixation (Meunier et al., 1997; see also Supplementary Figure 4), which, based on the O_2_ signal, seems to primarily occur during the first half of the dark period. The highest oxygen consumption occurs between D3 and D4, which is congruent with both the minimum O_2_ evolution and the maximum respiration rate also reported around D4 in the same species by Sherman et al. (1998). Thus, the change in OD_720_ is caused by respiration of refractile carbohydrate storage granules to fuel N_2_-fixation. In good agreement with the O_2_ signal, OD_720_ then stabilizes at its minimum value over the last four hours of darkness, indicating that carbohydrate reserves are no longer being rapidly respired and N_2_-fixation activity terminated, causing the O_2_ concentration to increase and stabilize at a level comparable to the NO_3_^–^ culture (dotted line, Figures 2A,B). Comparison of these two dynamics suggests that no significant N_2_-fixation occurred in the NO_3_^–^ culture. Overall, the N_2_-fixation relies on the provision of ATP and, hence, on significant respiratory activity, while NO_3_^–^ uptake requires more electrons, but can proceed under illumination and, therefore, has less immediate effect upon O_2_ dynamics during the dark period. Given that respiration is O_2_-saturated at levels far below the concentration of O_2_ in air saturated water, we believe that O_2_ was not limiting metabolic processes at night. Had nitrogenase activity been limited by oxygen for respiration, a plateau would have been observed once the lowest O_2_ level was reached, instead of a negative peak (Figure 2B). Instead, the O_2_ signal did not remain at this minimum but re-increased before the 4^th^ hour of dark, indicating a decreased oxygen demand coincident with a decrease in nitrogenase activity. The temporal dynamics of nitrogenase activity suggested by the O_2_ signal is also further supported by the F_*M*_ and Y_(II)_ dynamics at night (Figures 3B,D), as capacity for electron transport around PSII has proved to be a proxy for nitrogenase activity in the unicellular diazotroph *Cyanothece* (Rabouille et al., 2014).

The incorporation of labelled C and N presented in a companion paper clearly indicates that N2-fixation occurs primarily in the first half of the dark period, and decreases by two orders of magnitude during the second half of the dark phase (1.34 ± 0.79 d^–1^ in early night vs. 0.012 ± 0.047 d^–1^ in late night; Polerecky et al., 2021). This result is in good agreement with the OD_720_ dynamics, confirming that the active respiration of carbohydrates to fuel N_2_-fixation also stops by the mid-dark phase. However, cells still contain significant bulk polysaccharide reserves at the end of the dark phase (see D10 = L0 in Tables 1, 2), indicating that termination of N_2_-fixation is not due to exhaustion of the carbon reserves potentially available to fuel this activity. The nitrogenase enzyme is under the control of a tight circadian program which probably triggered a post translational regulation of and/or decay of the enzyme pool (Cólon-López et al., 1997). We postulate that the reason for such a control before the end of the dark is related to the replenishment of the nitrogen reserves: sufficient new N_2_ may have been assimilated into N storage given the overall growth rate achieved under the present conditions, making it unnecessary for the N_2_-fixation machinery to keep operating.

### Photosynthetic Efficiency

Similarly to other oxygenic photoautotrophs, cyanobacteria feature an excitation energy transfer from the antennae to photosynthetic reaction centres and the distribution of absorbed light between photosystems that can be modulated by short-term light acclimation processes. In particular, state transitions balance the distribution of excitation energy between photosystems, depending upon the redox state of the plastoquinone (PQ) pool (Meunier et al., 1997). These regulatory processes interact to cause changes in photochemical and Chl *a* fluorescence yields depending upon light and metabolic conditions. In the following, we used the fluorescence data to reveal the mechanisms underlying the observed two phases during the light period.

**FIGURE 4:**
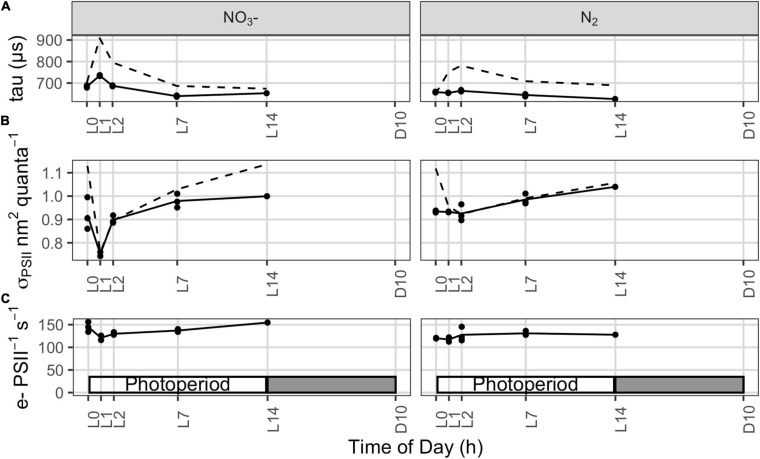
**(A)** Lifetime for reopening of Photosystem II, τ (μs), reflecting capacity for transport of electrons away from PSII, measured using FRRf relaxation after a single turnover saturating flash, applied on top of actinic illumination (τ′, solid lines), similar to the growth light level of 300 μmol photons m^–2^ s^–1^ in the photobioreactors. Dashed lines show the average of τ measures taken from the same samples in darkness. **(B)** Effective absorption cross section for Photosystem II measured using FRRf under growth illumination (solid lines; σ_*PSII*_′, nm^2^ quanta^–1^) or under darkness (dashed lines; σ_*PSII*_, nm^2^ quanta^–1^). **(C)** Photosystem II electron transport (e^–^ PSII^–1^ s^–1^) under growth illumination estimated from FRRf parameters, following eqn.10.

Online fluorescence monitoring within the cultures was used to probe the efficiency of PSII (Y_*II*_), which is a proxy for the immediate conversion of photon energy into chemical energy. Under favorable conditions, the expression of genes encoding PSII subunits is highest in the very early light phase (Stöckel et al., 2008), suggesting that PSII should quickly be operational upon the onset of the light. In the early light phase, the F_*t*_ signal closely approaches F_*M*_′ in the NO_3_^–^ cultures (Figure 3A), which leads to a drop in Y_*II*_ upon illumination (Figure 3C). This is indicative of a stronger PSII closure compared to the N_2_-fixing culture (Figures 3B,D), consistent with the higher phycobiliprotein content (see Supplementary Material) allowing a broader collection of photons, and therefore higher excitation pressure upon PSII, in the NO_3_^–^ cultures. The observed, lower photosynthetic yield in the NO_3_^–^ cultures coincides with a lower rate of carbon incorporation and change in optical density (Figure 1) in the early light phase. This Y_*II*_ pattern is also further confirmed by longer lifetimes (τ) for PSII reopening after a single turnover saturating flash (Figure 4A, solid line), particularly in τ measured immediately after a shift to darkness (Figure 4A, dashed line). These Y_*II*_ and τ dynamics suggest that PSII reopening is slowed down because electrons linger downstream: they are not very efficiently drawn away from PSII, which then tends to remain closed for longer times. This downstream limitation upon electron transport away from PSII relaxes later in the photoperiod (from L4 in the NO_3_^–^ cultures and L3 in the N_2_-fixing cultures) when the τ from NO_3_^–^ and N_2_-fixing cultures converge under DIC limitation, allowing F_*t*_ to again fall below F_*M*_ Figures 3A,B) as the PSII pool partially reopens. We thus have the counterintuitive finding that capacity for electron transport away from PSII actually accelerates as the cells progress from a DIC replete light phase of rapid light-saturated oxygen evolution to DIC limited oxygen evolution. This acceleration of downstream electron transport capacity reflects opening of electron fluxes, which is also paralleled by a partial reestablishment of growth (Figure 1) during the photoperiod. Fluorescence data therefore further support the conclusion that CCM is induced in both culture treatments under DIC limited growth (Badger and Price, 2003) and generates additional electron flux downstream of PSII.

During the initial light phase with intense oxygen evolution, the effective absorption cross section for PSII under growth irradiance (σ_*PSII*_′, nm^2^ quanta^–1^) is transiently down-regulated in the NO_3_^–^ cultures (Figure 4B), consistent with the quenching of fluorescence yields observed over the same period from the built in sensors in the photobioreactors (Figure 3A). These findings, together, suggest a state transition towards State II, with a transient drop in relative allocation of excitation to PSII photochemistry. These changes in fluorescence yield and σ_*PSII*_′ could possibly also be explained by induction of fluorescence quenching through the Orange Carotenoid Protein (OCP) system (Kirilovsky, 2015), which is encoded in the *Cyanothece* genome. But the parallel increase in τ (Figure 4A) rather indicates changes in downstream processes which lead to an increased reduction of the intersystem electron carriers, triggering the transition towards State II. Furthermore, the built in detectors show parallel responses of fluorescence whether excited either by blue, through Chl *a*, or by orange/red, through the phycobilisome antenna (data not shown). A response mediated by OCP would rather be expected to act preferentially upon excitation delivered through the phycobilisomes, rather than similarly upon excitation delivered through both Chl *a* and the phycobilisomes.

In contrast, this initial decline in σ_*PSII*_′ and increase in τ upon illumination are absent or muted in the N_2_ bioreactors (Figure 4B), perhaps reflecting differences in electron dynamics related to the depletion of carbohydrate reserves in darkness in the N_2_-fixing cultures, compared to the NO_3_^–^ cultures which retain carbohydrate reserves from the dark phase to support respiration upon re-illumination. Furthermore, the phycobilisome content (both allophycocyanin and phycocyanin) is lower in the N_2_-fixing cultures (Tables 1, 2; see Supplementary Material), possibly affecting state transition and excitation pressure upon PSII.

Upon the end of the light saturated phase, the NO_3_^–^ cultures transition back towards State I as shown by the increase in σ_*PSII^′*_ (Figure 4B). This, together with the recovery of fluorescence yields within the culture (Figure 3A) and a progressive decrease in τ (Figure 4A), suggest the induction of down-stream capacity to carry electrons away from PSII allowing re-oxidation of the intersystem transport chain.

Even though net oxygen evolution is high during early illumination (Figure 1), PSII-mediated electron transport (e^–^ PSII^–1^ s^–1^), estimated from FRRf under growth illumination, decreases (NO_3_^–^ cultures) or remains steady (N_2_-fixing cultures; Figure 4C), consistent with the fluorescence quenching and down-stream limitation of electron transport observed over the same initial illumination period. This shows that over this period, despite slower electron flow through PSII, cells are nevertheless allocating a larger fraction of electrons flowing from PSII to net assimilatory reduction, leaving a high net oxygen evolution.

When net oxygen evolution decreases to a lower rate limited by DIC (Figure 1), PSII-mediated electron transport (e^–^ PSII^–1^ s^–1^) actually increases (Figure 4C), consistent with an increase in pseudo-cyclic flow of electrons away from PSII but back to O_2_, as confirmed by MIMS measurements showing high rates of O_2_ uptake in the light reaching 37 ± 8% (N_2_-fixing culture) or 61 ± 16% (NO_3_^–^ culture) of gross O_2_ evolution (Supplementary Figure 5), thereby accelerating τ (Figure 4A) and allowing F_*t*_ to drop below F_*M*_′ as PSII centers re-open (Figure 3A). This re-opening is a gradual process from ∼L4.5 to ∼L9 in both the NO_3_^–^ and N_2_ bioreactors (Figures 3, 4A), consistent with a regulatory induction of alternate electron sink(s) concurrent with the onset of DIC limitation of oxygen evolution (Figure 1). This induction, however, does not depend upon DIC limitation because a similar pattern occurred even in bioreactors bubbled with CO_2_ to relieve the diel onset of DIC limitation (Supplementary Figure 1). Taken together, the data show a constitutive induction of alternate electron flows in late subjective morning, possibly after cellular requirements for CH_2_O accumulation have been met or saturated during the initial period of rapid net oxygen evolution (Polerecky et al., 2021).

Finally, upon the onset of darkness, PSII centers open (increasing F_*M*_ signal relative to F_*t*_, Figures 3A,B), Y_*II*_ thereby increases (Figures 3C,D) and the potential for PSII-mediated electron transport fully recovers in the NO_3_^–^ cultures or remains steady in the N_2_-fixing cultures (Figure 4C).

**FIGURE 5:**
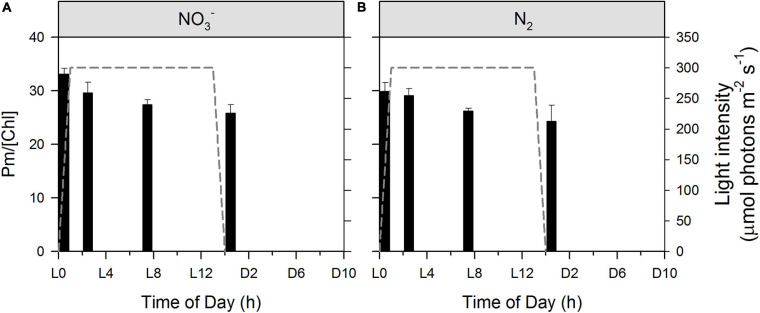
Dynamics of relative PSI abundance during *Cyanothece* cultivation in ASP 2 medium supplemented with NO_3_^–^ **(A)** and under obligate diazotrophy **(B)**. Maximal P_700_ levels (Pm), which is proportional to PSI abundance was normalized to corresponding Chl *a* concentrations. Data represent averages from 3 replicates, with error bars representing standard deviations. An ANOVA with *post hoc* Tukey HSD test points to i/a significant difference between L0 and L14 samples in both cultures and ii/no significant difference between cultures.

In agreement with 77 K fluorescence emission data showing a decrease in the PSI:PSII ratio during the light period (Supplementary Figures 2A,B), the maximal P_700_ level, Pm (normalized to Chl *a*), which is considered to be proportional to PSI abundance, decreases monotonically through the photoperiod in both the NO_3_^–^ and N_2_-fixing cultures, (Figures 5A,B). As Chl *a* is associated with both PSII and PSI, a decrease in PSI abundance relative to Chl *a* suggests an increase in the PSII population, which is consistent with a decrease in quantum yields of non-photochemical energy dissipation in PSI due to donor side limitation, Y(ND) (data not shown). Since there are more PSI than PSII in the cells, the increase in PSII abundance must be more pronounced than the decrease in the (relative) PSI abundance. Thus, the opening of PSII reaction centers and upregulation of the linear electron flow to alternate electron sinks during the light phase (see Figure 4 and corresponding text) are accompanied by a concomitant increase in the PSII to PSI ratios and these processes take place in a concerted manner. Similar phenomena were found during the diel cycle of *Crocosphaera watsonii* WH8501 (Masuda et al., 2018), with a further inactivation of PSII in the dark (Rabouille and Claquin, 2016), and in greening *Synechocyctis* sp. 6803 (Barthel et al., 2013). The observed transition from State II back to State I later in the photoperiod (Figure 4) also supports an enhanced flux of electrons to alternate electron sinks. In accordance, again, with the 77 K fluorescence emission data (Supplementary Figures 2A,B), no significant difference is observed between the NO_3_^–^ and N_2_-fixing culture.

**FIGURE 6:**
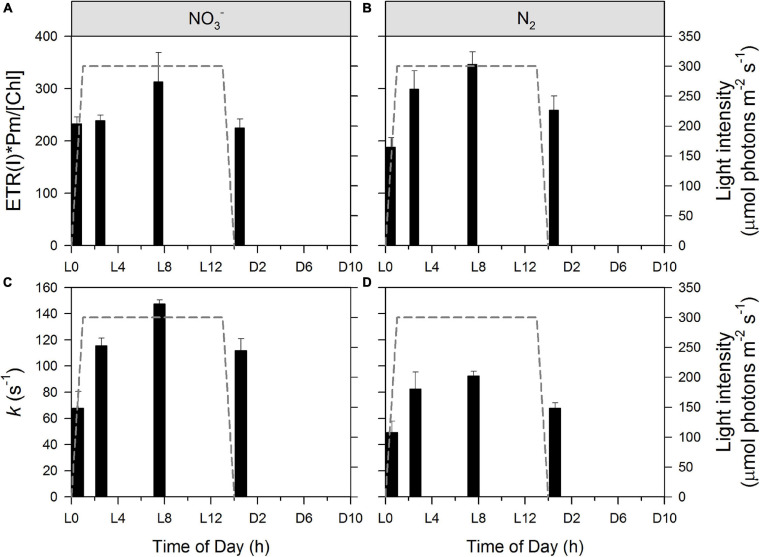
Dynamics of PSI-mediated electron flow [ETR(I)] during *Cyanothece* cultivation in ASP 2 medium supplemented with NO_3_^–^ **(A,C)** and under obligate diazotrophy **(B,D)**. Raw ETR(I) values were normalized according to Pm values and Chl *a* concentration **(A,B)**. k represents the rate constant for re-reduction of P_700_ by intersystem electrons after a flash **(C,D)**. Data represent averages from 3 replicates, with error bars representing standard deviations.

Kinetics of electron transport processes through and around PSI were determined by P_700_ SP methods. PSI-mediated electron flow [ETR(I)] shows a characteristic daily pattern in both the NO_3_^–^ and N_2_-fixing cultures (Figures 6A,B, respectively). An ANOVA with *post hoc* Tukey HSD test (n = 3 at each column) indicates that values do not differ significantly in statistical terms in the NO_3_^–^ culture (Figure 6A), while L0 differs significantly from both L2 (p < 0.05) and L7 (p < 0.01) in the N_2_-fixing culture (Figure 6B). However, much stronger statistical differences appear in the dynamics of *k*, the rate constant for re-reduction of P_700_ by intersystem electrons after a flash. L0 significantly differs from all other time points in the NO_3_^–^ culture and from L2 and L7 in the N_2_-fixing culture. There is an additional statistically significant difference between the NO_3_^–^ and N_2_-fixing cultures at L14 = D0 (Figure 6B). Quantum yields of PSI [Y_(I)_] show very similar patterns (data not shown). ETR(I) and Y_(I)_ are relatively low in both types of cultures at D0 (light to dark transition) and L0 (dark to light transition) and then show a gradual increase to a daily maximum during the light phase (L7). This increase is in fairly good correlation with the online records of the photobioreactors showing partial re-opening of PSII (Figure 3) and PSII functional data (Figure 4B) showing an acceleration of electron transport away from PSII. The increase is slower in the NO_3_^–^ culture (values at L0 and L2 are almost the same) and faster in the N_2_-fixing culture. This suggests that the increase in ETR(I) is (directly or indirectly) related to the kinetics of O_2_ and pH (i.e. drawdown of the DIC pool), and possibly also State I to State II transition, which all show similar differences between NO_3_^–^ and N_2_-fixing cultures (Figures 2A–D). Remarkably, the pH increase in the cultures results in a faster ETR(I) in chloroplasts (Tikhonov, 2013 and references therein), in good agreement with our finding. It was postulated that alkalization of stroma (in cyanobacteria: alkalization of cytoplasm) induces activation of the Bassham-Benson-Calvin cycle reactions, and, thereby, promotes efflux of electrons from PSI to NADPH^+^ (Tikhonov, 2013). Concomitantly, the increase in ETR(I) could also be directly related to the induction of CCM (i.e., cyclic electron transport around PSI and the NDH-1 complex for CO_2_ uptake, see Supplementary Figure 6 and text above), which could also explain the higher ETR(I) in N_2_-fixing culture (compared to NO_3_^–^ culture) at L2, when N_2_-fixing culture is already DIC depleted, while NO_3_^–^ culture still has some DIC (Figures 2E,F) available. A similar daily pattern of an increase from L0 to daily maximum (L7), was observed also in the rate of P_700_ re-reduction kinetics (Figures 6C,D) which show about a twofold increase in both the NO_3_^–^ and N_2_-fixing cultures. Nevertheless, there are remarkable differences in kinetics between these two growth conditions: rate constants (*k*) of P_700_ re-reduction kinetics increase from 68 ± 13 s^–1^ to 147 ± 3 s^–1^ in NO_3_^–^ culture but only from 49 ± 9 s^–1^ and 92 ± 4 s^–1^ in the N_2_-fixing culture. This, again, can partly be explained by a difference in external pH (Figures 2C,D) which was lower in the N_2_-fixing culture, and, therefore, implied slower P_700_ re-reduction kinetics (Tikhonov, 2013). Importantly, the observed increase in ETR(I) (Figures 6A,B), rate of P_700_ re-reduction kinetics (Figures 6C,D), and the State II to State I transition (Figure 4) during the light phase perfectly complement the re-opening of the PSII RCs and acceleration of τ showing again that all of these processes take place in a concerted manner.

**FIGURE 7:**
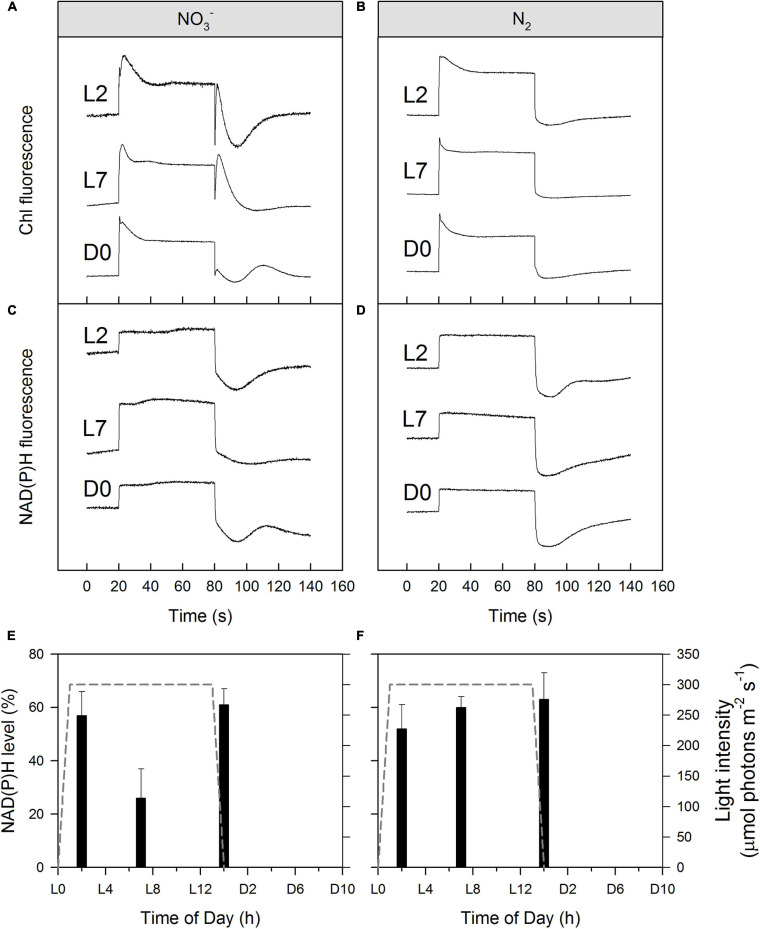
Simultaneously recorded Chl *a* **(A,B)** and NAD(P)H fluorescence **(C,D)** induction curves (with recovery) of *Cyanothece* cultures during/after the light phase as well as corresponding calculated NAD(P)H levels as a percentage of the entire NAD(P)H:NADP^+^ pool in darkness **(E,F)**. Cultures were nitrate-rich **(A,C,E)** or under obligate diazotrophic conditions **(B,D,F)**. Panels **(A–D)** show typical induction curves, while data on panels **(E,F)** represent averages from 3 to 4 replicates, with error bars representing standard deviations. For more details, see Materials and Methods.

NAD(P)H is a reducing agent and an essential electron donor in all organisms. The rate at which it regenerates from its oxidized form (NAD(P)^+^) can therefore constitute a limiting factor in different biosynthetic pathways. The bioenergetic costs of diazotrophic (N_2_) growth versus reductive assimilation of NO_3_^–^ in *Cyanothece* show a major difference in ATP vs. NAD(P)H demand of the NO_3_^–^ and diazotrophic (N_2_) cultures, due to the high electron demand of NO_3_^–^ uptake (8 + 2 e^–^/N) relative to N_2_ reduction (1—2 e^–^/N) (see also Introduction). Hence, remarkably higher NAD(P)H/ATP ratios are needed in the NO_3_^–^ cultures as compared to diazotrophic (N_2_) cultures, and this higher demand for reductant must have a major influence on electron transport and NAD(P)H dynamics as well. High rates of electron transport to O_2_ via the photosynthetic electron transport chain (e.g., Mehler reaction, photorespiration, flavodiiron protein-dependent O_2_ uptake; Supplementary Figure 5) lead to an increased reduction of the PQ-pool, which, in turn, triggers a transition towards State II (Figure 4). The lower respiratory activity and accompanying high accumulation of carbohydrates during the light period in the N_2_-fixing culture (Tables 1, 2 and Supplementary Figure 5) are in good agreement with both the lower overall demand for reductants and the high nocturnal energy demand related to dark N_2_-fixation in this culture. These differences in the demand for reductants and consequently in respiratory electron fluxes, are also shown in the post-illumination pattern of the Chl *a* fluorescence. A significant post-illumination reduction of the PQ pool is seen in the NO_3_^–^ culture during its photoperiod (Figure 7A, L2 and L7 at 80s). In contrast, this post-illumination fluorescence transient is much smaller in the corresponding D0 (= L14) sample and is completely absent in all records of the diazotrophic (N_2_) culture (Figures 7A,B).

Regarding NAD(P)H/NAD(P)^+^ redox levels, in most of the NO_3_^–^ and N_2_-fixing culture samples we observed about 50–60% reduction in the dark-adapted samples (Figures 7C–F), in good agreement with the literature range (Kauny and Sétif, 2014). However, in the midday (L7) NO_3_^–^ cultures (Figures 7C,E, 7 h) the NAD(P)H/NAD(P)^+^ redox level was only 26 ± 11% indicating an enhanced NAD(P)H consumption in these samples, again, in accordance with the high e^–^ demand of NO_3_^–^ to NH_4_^+^ reduction (see above) and with our hypothesized opening of alternate electron sinks upon drawdown of the DIC pool.

### Growth Budgets

The transient storage of reducing potential as carbohydrate imposes additional losses compared to a hypothetical N_2_ assimilation directly driven by photosynthetic electron transport. Fueling N_2_-fixation at night requires first prior carbon fixation (equations 3 and 4) and then carbohydrate re-oxidation (equation 8) through aerobic respiration (equation 9), to produce both the ATP and reducing power needed to fix N_2_.

The electrons produced by carbohydrate re-oxidation in the dark must be partitioned between ATP generation, or biosynthetic reductions, as for example:

(13) N+4e+-8ATP→→NH+4+1/2H+28ADP+8Pi

The fixation of one mole of N thus requires 1 CH_2_O worth of biosynthetic reductant (4e^–^) and 1.33 CH_2_O (6e^–^) worth of reductant for the respiratory regeneration of the required ∼ 8 ATP, so in total the equivalent of ∼2.33 stored CH_2_O, a slightly higher estimate than provided by Großkopf and LaRoche (2012), who counted a requirement of 3e^–^ for N_2_-fixation and the equivalent 1.33 CH_2_O to generate the required 8 ATP. Following up the stoichiometric analyses presented in the introduction, for a cell with C7:N1 ratio growing diazotrophically with CO_2_ fixation in the light period and N fixation in the dark period at the expense of previously stored carbohydrate:

Light period:

{7Cnet+2.33Cstored}×{CO+2external4e+-4ATP→CHO2+4ADP+4P}i=37.3e-+37.3ATPconsumed

Dark period:

{2.33Cstored}×{CHO2→→CO+24e}-=9.3e-

consumed(4eallocated-toreductiveassimilationand5.33efor-respiration)

5.33e×-{O+26ADP+6P→i2HO2+6ATP}/4e=-8⁢A⁢T⁢P⁢produced

**FIGURE 8:**
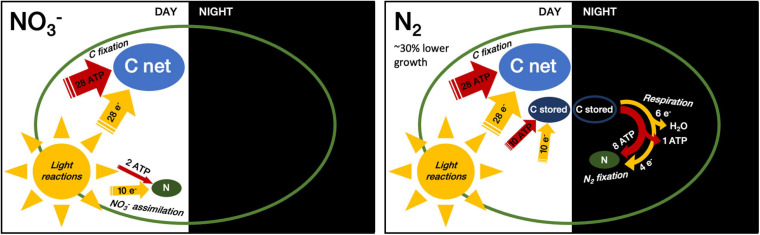
Relative consumption of energy equivalents by C and N assimilation in NO_3_^–^ consuming vs. N_2_ fixing cells. Width of arrows and size of pools reflect approximate relative amounts of energy equivalents (ATP and reducing equivalents) and pool sizes (C net, C stored and N) per mole of assimilated N. This figure compares direct assimilation costs for C and N, ignoring basal respiration as well as indirect costs such as those for daily *de novo* synthesis of nitrogenase. While carbohydrate accumulation occurs in both N conditions, N_2_-fixation requires the build-up of an additional, transient C pool (C stored) that is respired to fuel N_2_-fixation during the following night.

Overall the cells using N_2_-fixation thus incur a daily cost of 38 e^–^ + 38 ATP per assimilation of C7:N1. In contrast, as outlined in the **Introduction** a cell with a C:N-ratio of 7:1, growing upon NO_3_^–^ with direct photosynthetic generation of biomass implies an allocation of 38 e^–^ and 30 ATP overall (Figure 8). This opportunity cost of ∼8 additional ATP for offsetting dark N_2_-fixation from light CO_2_ fixation thereby contributes to the lower growth rate we observed in the N_2_-fixing cultures. In the end, this would imply a 8/30 = 27% increase in diel ATP costs for diazotrophic growth. Although overly simplistic, this analysis is in good agreement with the achieved diel growth rates of μ_*NO*__3_ = 0.312 d^–1^ vs. μ_*N*__2_ = 0.216 d^–1^, which point to a 31% drop in the overall growth efficiency under diazotrophy. That is, the observed difference in the net growth rate matches the direct penalty to grow on N_2_-fixation. Yet, the energy needed to synthesize the nitrogenase enzyme and the enhanced respiration required to lower oxygen concentration within cells impose additional costs that could further affect the growth efficiency. Because the resultant, net growth in the N_2_-fixing cultures wasn’t impacted any further, we deduce that the larger carbon reserves accumulated in the N_2_-fixing cultures were sufficient to cover all the indirect costs. It is very likely that, had cultures been grown under replete CO_2_, the N_2_-fixing cells would have accumulated more reserves, thus possibly allowing a better coverage of the direct costs of N_2_-fixation as well. As stressed by Großkopf and LaRoche (2012), the energetic investment associated with assimilatory NO_3_^–^ reduction and N_2_-fixation (when considering the direct costs only) can come rather close, and depend on the respiration efficiency (ATP generated per CH_2_O respired).

## Conclusion

When grown on NO_3_^–^, *Cyanothece* uses the photoperiod for simultaneous, reductive assimilation of both CO_2_ and NO_3_^–^ with an overall metabolic e^–^:ATP stoichiometry requirement close to the predicted output of linear photosynthetic electron transport. In this mode, direct reductive NO_3_^–^ assimilation competes with inorganic carbon assimilation for photosynthetic reductant, which slows the drawdown of the DIC pool in our turbidostat experiments. The subsequent dark phase is then a period of maintenance respiration and synthesis of cell components like Chl *a*, and proteins (Polerecky et al., 2021). In marked contrast, *Cyanothece* grown in obligate diazotrophy does not take up nitrogen in the light and therefore can funnel more photosynthetic reductant to CO_2_ assimilation, thereby accumulating a larger stock of refractile carbohydrate, a necessity to subsequently support the e^–^:ATP requirements of N_2_-fixation during the succeeding dark period. Consequent to the higher rate of carbon fixation in the early light is a more rapid depletion of available DIC, explaining why DIC becomes limiting earlier in the day in the N_2_-fixing cultures. In the early dark period, a high respiration rate engages to fuel N_2_-fixation; later on in the dark, and despite still available carbohydrate reserves, *Cyanothece* reverts to a dark maintenance mode similar to that observed in the NO_3_^–^ cultures.

In our experiments, *Cyanothece* achieves higher overall growth rates under NO_3_^–^. When grown in obligate diazotrophy, cells face the double burden of a more ATP-demanding, N-acquisition mode and the additional losses imposed by the transient storage of reducing potential as carbohydrate, compared to a hypothetical N_2_ assimilation directly driven by photosynthetic electron transport. Further, this energetic burden imposed by N_2_-fixation could not be alleviated, despite the high irradiance level within the cultures, because photosynthesis was limited by the availability of DIC. A relaxation of DIC limitation in the bioreactors might allow N_2_-fixing *Cyanothece* to accelerate towards the growth rate of NO_3_^–^ *Cyanothece*, by unleashing a higher carbon fixation potential in the light when growing diazotrophically. But, bearing in mind that the capacity of cells to store reserves is also sterically limited (Talmy et al., 2014), whether saturating light and DIC levels could allow the obligate diazotroph to match the growth rates of the nitrate-grown *Cyanothece* still has to be demonstrated.

The higher instantaneous carbon fixation rates, consequent to the temporal decoupling between carbon and nitrogen acquisition under diazotrophy, probably constitute a competitive advantage in the natural environment, given the known, high storage capacity of unicellular diazotrophic cyanobacteria. Yet, as DIC limitation exacerbates the costs on growth imposed by N_2_-fixation, the competitive efficiency of such diazotrophs could be hindered in areas with insufficient renewal of dissolved gases and/or with intense phytoplankton biomass that both decrease available light energy and draw the DIC level down, like lakes and the coastal ocean during bloom periods. As progress is made in the automation of *in situ* instruments, the future literature shall inform on a closer monitoring of DIC availability at the diel scale and finer spatial scales that will reveal the likelihood and frequency of DIC limitation in coastal environments, for a better understanding of cyanobacterial dominance.

Lastly, this study also stresses how important it is to carefully monitor culture experiments. Undesired limitations or differences can easily and surreptitiously introduce a bias in the experiment and lead to potentially erroneous results. A difference in light level will occur in the culture as soon as either the control or treatment starts to get denser than the other. Light then becomes a co-limitation factor with differential impact on the cultures, leading to a distorted observation of the initially targeted, physiological response. DIC limitation is also a potential source of biases in culture experiments and our present results also serve to illustrate how much that can affect both immediate (e.g. photosynthesis) and temporally de-coupled (e.g. N_2_-fixation) processes and the overall growth response.

## Data Availability Statement

The raw data supporting the conclusions of this article will be made available by the authors, without undue reservation.

## Author Contributions

The experimental part of this study was conducted during the 10th Group for Aquatic Productivity (GAP) workshop in August 2017 organized by OP. SR, TM, DC, KH, JČ, and OP designed the study. All authors contributed to sampling, samples analyses and data interpretation. SR and DC drafted the manuscript and all authors provided input during writing of the manuscript.

## Conflict of Interest

The authors declare that the research was conducted in the absence of any commercial or financial relationships that could be construed as a potential conflict of interest.
