# Supplementary material for: Electron & Biomass Dynamics of Cyanothece Under Interacting Nitrogen & Carbon Limitations
Source: Front Microbiol. 2021 Apr 9;12:617802. doi: 10.3389/fmicb.2021.617802 (PMC8063122; doi:10.3389/fmicb.2021.617802)
Supplement: Supplementary file 1 [file Data_Sheet_1.pdf]

Supplementary Information

Electron & Biomass Dynamics of Cyanothece under interacting N & C limitations

Sophie Rabouille[1,2],
Douglas A. Campbell[3,4],
Takako Masuda[3]
Tomáš Zavřel[5],
Gábor Bernát[3,6],
Lubos Polerecky[7]
Kimberly Halsey[8]
Meri Eichner[3,9]
Eva Kotabová[3],
Susanne Stephan[10,11]
Martin Lukes[3],
Pascal Claquin[12],
José Bonomi-Barufi[13]
Ana Teresa Lombardi[14]
Jan Červený[5],
David J. Suggett[15],
Mario Giordano[16,3],
Jacco C. Kromkamp[17],
Ondřej Prášil[3]

[1]Sorbonne Université, CNRS, LOV, F-06230 Villefranche-sur-mer, France
[2]Sorbonne Université, CNRS, LOMIC, F-66650 Banyuls-sur-mer, France
[3]Centre Algatech, Institute of Microbiology of the Czech Academy of Sciences, Třeboň, Czechia

[4]Mount Allison University, New Brunswick, Canada

[5]Department of Adaptive Biotechnologies, Global Change Research Institute CAS, Brno, Czech Republic

[6]Centre for Ecological Research, Balaton Limnological Institute, Klebelsberg Kuno u. 3. 8237 Tihany, Hungary

[7]Department of Earth Sciences, Utrecht University, Utrecht, The Netherlands

[8]Department of Microbiology, Oregon State University, USA

[9]Max Planck Institute for Marine Microbiology, Bremen, Germany

[10]Leibniz-Institute of Freshwater Ecology and Inland Fisheries, Zur alten Fischerhütte 2, 16775 Stechlin, Germany

[11]Department of Ecology, Berlin Institute of Technology (TU Berlin), Ernst-Reuter-Platz 1, 10587 Berlin, Germany

[12]UFR des Sciences, Université de Caen-Normandie, France

[13]Departamento de Botânica, Centro de Ciências Biológicas, Universidade Federal de Santa Catarina, 88040-970, Florianópolis, SC, Brazil

[14]Universidade Federal de São Carlos, São Carlos, Brazil

[15]University of Technology Sydney, Climate Change Cluster, Faculty of Science, Ultimo, NSW, 2007, Australia

[16]Dipartimento di Scienze della Vita e dell'Ambiente, Università Politecnica delle Marche, Ancona, 60131, Italy

[17]NIOZ Royal Netherlands Institute for Sea Research and Utrecht University, The Netherlands

1. Culture setup, monitoring and control

Experimental design

Once cultures reached the desired cell density (up to 3 days after inoculation) the photobioreactors were switched to a turbidostat mode to give direct control of irradiance conditions within the cultures. Dilution cycles of 5% (v/v) with fresh media were activated each time the culture density reached a threshold level (of OD_{680} ~0.51). When peristaltic pumps were activated, a fixed pumping rate was used to dilute the culture below the threshold level (to OD_{680} ~0.49). Pumps supplied only fresh medium; culture outflow occurred when the culture volume reached its upper limit. The turbidostat mode allowed to maintain cultures at approximately constant cell densities of 1 - 1.4 × 10^7 cells mL^{-1} irrespective of their actual growth rate, in order to impose a similar irradiance level within all cultures. This was important to prevent any bias that might arise as a result of different irradiance conditions between treatments. A magnetic stirrer spinning in the cultures ensured homogeneity of the cultures, prevented sedimentation at the bottom of the reactor, and also, together with bubbling, drove renewal of dissolved gases in the medium.

Cultures were run in triplicates and sampling in each culture was repeated in time, for five days in a row. This resulted in a both instantaneous replication of sampling across bioreactors and temporal replication within each bioreactor. Values belonging to the same diel sampling time (L0, etc.) over the experimental window were considered as technical replicates (n=5), while values determined

at a particular time point using samples from different bioreactors (with identical growth conditions) were considered as biological replicates (n=3).

Samples for measurement of dissolved inorganic carbon (DIC) concentrations in the cultures were filtered through 0.2 μm syringe filters and stored at 4°C prior to use. DIC concentrations were determined with a custom-built flow injection system at the Max Planck Institute for Marine Microbiology (Bremen, Germany) according to Hall and Aller (1992). Briefly, a small volume of the sample (50 μL) was injected into a stream of 30 mmol L^{-1} HCl to convert all DIC to CO_2, which then diffused via a Teflon membrane into a 5 mmol L^{-1} NaOH receiver stream, where the formation of carbonate ions was detected as a change in conductivity. Four technical replicate measurements were performed on each sample.

2. Biomass buildup and transient growth dynamics

Biomass concentration and cellular, nitrogen- and carbon-rich pools

Cell count and cell size distribution were determined using a Multisizer 4 COULTER COUNTER® (Beckman Coulter Inc., Brea, CA, USA). Particulate organic carbon (POC) and nitrogen (PON) were analyzed using a CHN analyser (PerkinElmer PE2400, PerkinElmer Inc., Waltham, MA, USA). Samples (10 mL) were collected by centrifugation (28°C, 8000 rpm, 7 min), dried at 60°C, and wrapped into tin capsules prior to analysis. Cellular carbon (fmol-C $cell^{-1}$) and nitrogen (fmol-N $cell^{-1}$) content were deduced using the cell abundance measured concomitantly. Chl *a*, carotenoid and carbohydrate (polysaccharide) content were estimated following previously reported protocols (Zavřel et al., 2015; Zavřel et al., 2018). Cyanophycin cell content was quantified by Sakaguchi reaction (Messineo, 1966), after concentration of samples (30 mL) by centrifugation (28°C, 8000 rpm, 7 min). All the cellular contents were expressed per cell (Table 1) and per μm^3 (Table 2).

While the total carbon buildup in cells was similar at the daily scale in both treatments (Table 1), a clear difference appears in carbon fluxes. Carbon reserves show dynamics typical of *Cyanothece*, with a minimum content in cellular polysaccharides at the end of the dark phase and a maximum in the mid (N_2-fixing cultures) to late (NO_3^- cultures) light phase (Table 1). Carbon reserves are catabolized in the dark in both treatments, but they fluctuate twice as much in the N_2-fixing culture

Total nitrogen shows distinct dynamics with a net increase in the dark and early light phase in the N_2-fixing cultures and a net increase in the light phase in NO_3^- cultures. On average, NO_3^- cultures contain more nitrogen ($p<0.05$), allocating more to both pigments and reserves. Cells grown on NO_3^- contain 33.6 % more cellular Chl *a* (143.0 ±14.3 *vs*. 107.1 ±17.8 fg $cell^{-1}$) and 10.6 % more carotenoid (44.0 ±4.8 vs. 39.7 ±5.5) compared to cells grown on N_2 (Table 1). Chl *a* and carotenoids expressed per biovolume are still higher in NO_3^- grown cells ($p<0.05$; Table 2). The average cellular cyanophycin content is significantly higher in NO_3^- cultures (n=12, $p<0.01$; Table 1). The temporal dynamics of this pool also differs between treatments. It shows a high turnover rate in the N_2-fixing cultures, with an accumulation in the dark and the consumption of this pool in the light. This pool keeps increasing in the light in the NO_3^- cultures, most likely because NO_3^- uptake happens mainly in the light phase (Polerecky et al., *in press*), also reaching a higher level of storage. These results suggest that the turnover rate of cyanophycin is more than one day in the NO_3^- culture.

In the end, both differences in dynamics and amplitude lead to distinct C:N ratios, with wider fluctuations in the N_2-fixing cultures and an average value that is higher in the NO_3^- culture but still below the Redfield canonical value (Table 1).

The relative carbon allocation in proteins, carbohydrates, and lipids was estimated using Fourier Transform Infrared Spectroscopy (FTIR). Culture samples (2 mL) were pelleted by centrifugation at 3000*g* for 5 min at 4ºC and the supernatant was discarded. The pellet was washed with 1mL isosmotic solution (28 g L^{-1}) of ammonium-formate to remove salt while avoiding disruption of cells, and then centrifuged again. The pellet was resuspended in 50 µL of ammonium-formate solution and a sub-sample was taken for cell counts (Beckman Coulter III). An appropriate volume was then spread and re-diluted on an Si 384 well plate to get a final cell content of about $3 \cdot 10^5$ cells in a total volume of 10 µL in each well. The FTIR spectra were obtained using a Nicolet IS10 (Thermo Nicolet, Madison, WI, USA) spectrometer equipped with a microarray reader with a Deuterated Tri-Glycine Sulphate (DTGS) detector. Spectra were collected at a spectral resolution of 4 cm^{-1}; each spectrum represents the average of 64 scans on the same well and a minimum of 3 wells were read per culture sample to check for the internal variability of the sample. A Blackman-Harris three-term apodization function was used, with a zero-filling factor of 2. OMNIC software (Nicolet) was used for both measurement and data processing. IR bands were assigned according to Dean et al. (2008). Lipid & fatty acid, protein and carbohydrate content was determined based on the amplitude of the 1735 cm^{-1} band (ester C=O stretching), the two contiguous bands at around 1652 cm^{-1} (Amide I, C=O stretching of the peptide bond) and 1540 cm^{-1} (Amide II, combination of N-H bending and C-N stretching), and the 1152 cm^{-1} band (C-O-C stretching), respectively. As the cellular lipid content did not change during daytime or between treatments (data not shown), all spectra were normalized to the lipid band at ~1735 cm^{-1}. Proteins and carbohydrates were thus expressed as proportions relative to lipids, and the magnitude of peaks can be compared between spectra and treatments.

The total cellular nitrogen increases in the NO_3^- culture (Table 1) during the light phase, with NO_3^- primarily assimilated into cyanophycin (see companion paper by Polerecky et al *in press*). However, the protein content determined by FTIR is constant over the light period under NO_3^- (Fig. S1), suggesting net balance between protein synthesis and degradation in the light. On the other hand, the total cellular nitrogen of the diazotrophic culture even slightly decreases during the light phase (Table 1), confirming that N_2 fixation primarily occurs at night, as also indicated in Polerecky et al. (*in press*). At the beginning of the light phase, the protein content (determined by FTIR and normalised to total lipids, Fig. S1) of diazotrophic cells is significantly lower (by 30%) compared to NO_3^- cells. The protein content of the diazotrophic culture shows a clear and significant increase (by 16%) during the light phase (Fig. S1), indicating that protein synthesis from cyanophycin stored the preceding night exceeds protein degradation during the light phase. At the end of the light phase, the protein content of both cultures is similar.

Contrary to proteins, carbohydrates dynamics were very different both in terms of the amplitude of variations and temporal dynamics (Fig. S1). Results reveal an increase in cellular carbohydrates during the light and degradation in the dark in both conditions, which is typical under light:dark conditions in all cyanobacteria. But while this dynamic is moderate in the NO_3^- culture, the N_2-fixing culture shows a much higher carbohydrate build up in the light and their more pronounced consumption in the dark, clearly pointing to a strategy of diel carbon storage in the N_2-fixing cultures.

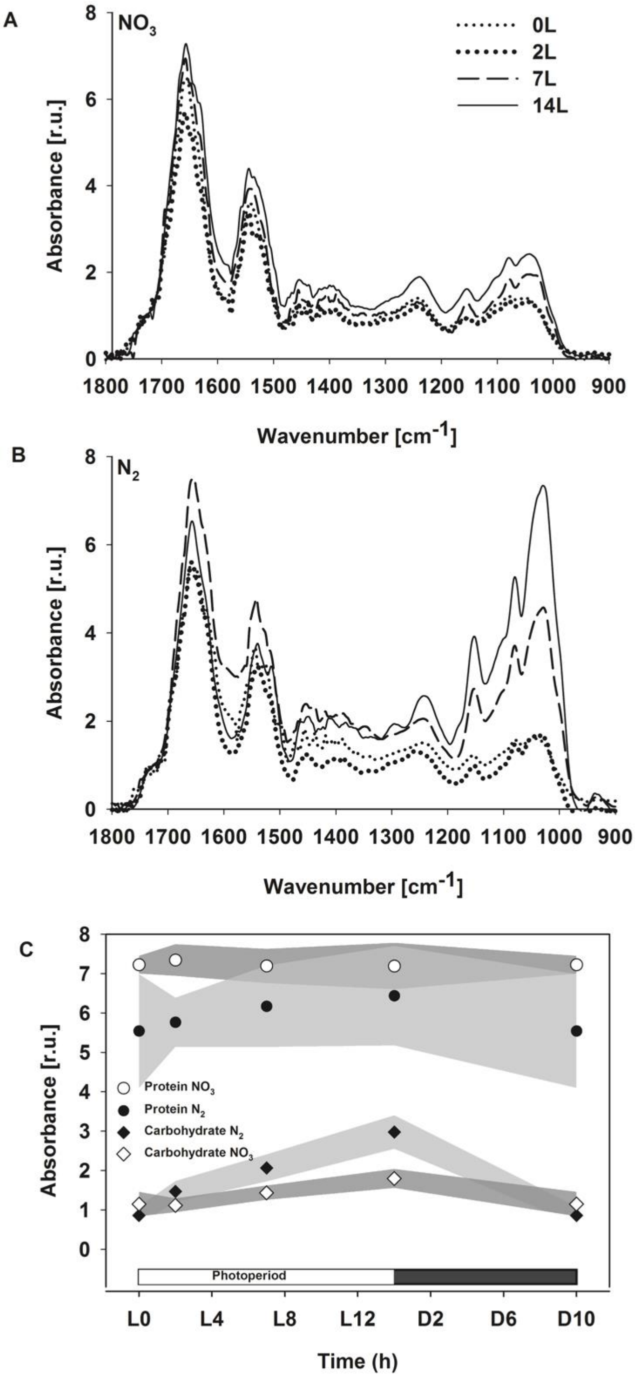

Figure S1. Representative FTIR spectra (A,B) and changes in time in the absorbance of the bands specific to amides (circles) and carbohydrates (diamonds) (C) of *Cyanothece* grown in ASP 2 medium supplemented with NO_3^- (A,C; dark grey) or under obligate diazotrophy (B,C; light grey), at a different time of a dark/light phase: L0 (soft dotted line), L2 (bold dotted line), L7 (dashed line) and L14 (solid line). The FTIR spectra were normalized to the maximum of υ(C=O) stretch of ester groups of lipids (1735 cm^{-1}). Lipid & fatty acid correspond to the band at 1735 cm^{-1}, proteins to the two contiguous bands at around 1652 cm^{-1} and 1540 cm^{-1}, while carbohydrates correspond to the 1152 cm^{-1} band .

Phycobilisome, Photosystem I and Photosystem abundance

Phycobilisomes composed of phycobiliproteins play a central role in cyanobacterial photosynthesis, both as the major light harvesting antenna (Gantt, 1980; Grossman et al., 2001), and also as potential reserves of amino acids. Phycobilisome abundance was derived from spectroscopy measurements using a Unicam UV500 scanning spectrophotometer (Thermo Spectronic, Cambridge, UK) equipped with an integrating sphere. 2 mL of cultures were filtered through GF/F filters and absorbance spectra recorded from samples on the filters. Spectra were then deconvoluted by PeakFit® software (Systat Software Inc., San Jose, California, USA) to clearly distinguish absorption peaks of chlorophyll *a* and phycobilisomes. The absorbances of phycobilisome peaks at 615 nm and 652 nm as well as of the residues at 720 nm were read using GetData Graph Digitizer software and corrected with corresponding β (i.e. correction) factors to account for scattering by the filter, derived from comparative measurements using identical samples both on filters and as cell suspensions in standard 1 cm quartz cuvettes: 615 nm: $\beta = 4.29$; 652 nm: $\beta = 4.54$; 720 nm: $\beta = 3.73$. After absorbance correction, the phycobiliprotein concentration was calculated according to Bennett and Bogorad (1973) and values are reported in Tables 1 and 2:

$$\text{Phycocyanin [mg mL}^{-1}] = [(A615 - A720) - 0.474 \times (A652 - A720)] / 5.34$$
$$\text{Allophycocyanin [mg mL}^{-1}] = [(A652 - A720) - 0.208 \times (A615 - A720)] / 5.09$$

Phycobiliprotein levels increased between L0 - L2, decreased between L2 - L7 and increased again between L7 - L14. The absolute values were lower in N_2-fixing cultures (varying between 497 ± 170 fg/cell to 621 ± 86 fg/cell) compared to NO_3^- cultures (685 ± 357 fg/cell to 743 ± 410 fg/cell at L7), consistent with the lower protein content in the N_2-fixing cultures.

The relative abundance of phycobilisomes and photosystems was further analysed via low temperature (77K) fluorescence emission spectroscopy (Fig. S2). An aliquot of 2 mL culture sample was taken at time points L0, L2, L7 and D0 (see. 2.1.) and gently filtered through a 0.4 µm glass fiber filter (GF-5, Macherey-Nagel, Düren, Germany). A part of the filter was cut out using an oval shaped hole puncher, placed into a copper sample holder and cooled down to 77K in liquid nitrogen in a Dewar flask with a transparent finger, designed for low temperature fluorescence emission measurements. Fluorescence emission spectra were recorded using an SM 9000 fluorimeter (Photon Systems Instruments, Drásov, Czech Republic) at excitation wavelengths of 455 nm (for Chl excitation) and 590 nm (for phycobilin excitation). Blank spectra were obtained using a moist filter and were subtracted from the raw spectra. A total of 20 spectra were recorded from the cultures at the different time points. All fluorescence spectra acquired in a given culture at a given time point were averaged (n=2 to 3). The baseline corrected fluorescence spectra were normalized to the 695 nm peak belonging to Photosystem II (PS II).

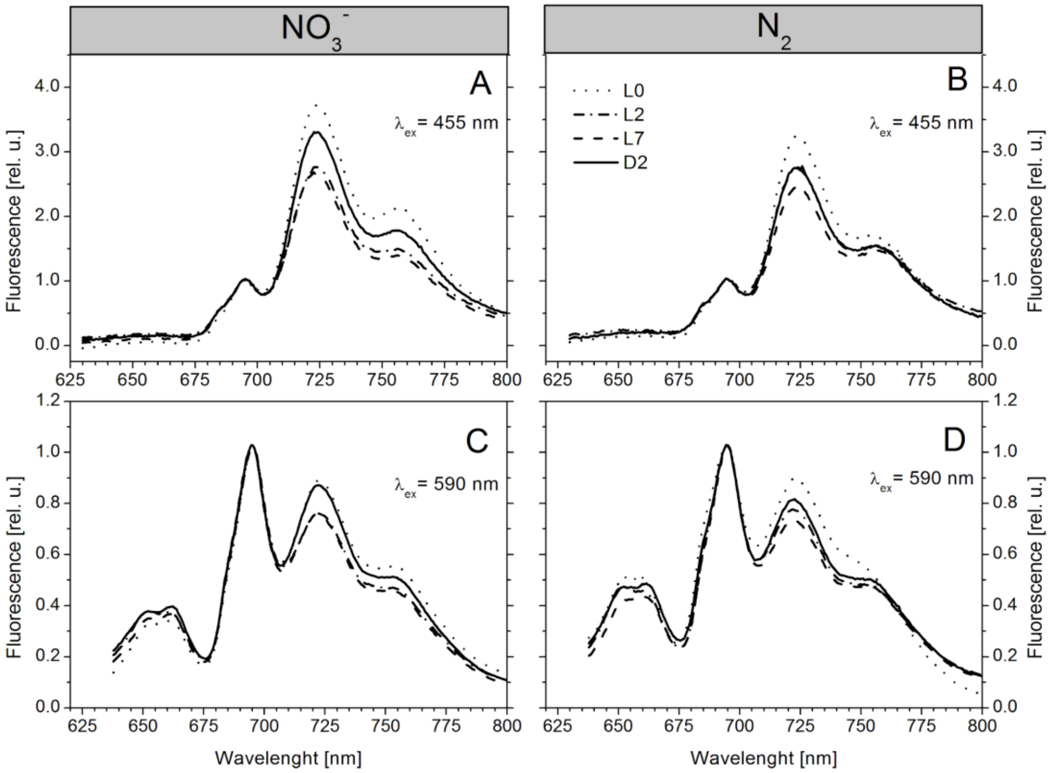

Figure S2. 77K fluorescence emission spectra excited at 455 nm (A, B) and 590 nm (C, D) of *Cyanothece* grown in ASP 2 medium supplemented with NO_3^- (A, C) or under obligate diazotrophy (B, D) at a different time of a dark/light phase: L0 (dotted line), L2 (dott-dashed line), L7 (dashed line) and D2 (solid line). Each fluorescence spectrum was normalized to the PSII-related emission maximum at 695 nm.

455 nm light primarily excites Chl *a* molecules within photosystems, while 590 nm light preferentially excites phycobilins. Therefore, the former spectra provides information on photosystem stoichiometry (Fig, S2, A,B), whereas the latter reveals relative phycobilisome abundance (Fig, S2, C,D). *Cyanothece* exhibits typical cyanobacterial fluorescence emission spectra with a 725 nm emission band of PSI, the 685-695 nm emission bands of the PSII core, and the phycobilisomes emission bands at 650-662 nm. In addition, the broad emission band centered at around 755 nm represents Chl vibronic sublevels. The PSI fluorescence emission compared to that of PSII was the highest at the onset of the light (L0) and decreased gradually during the light period in both the NO_3^- and N_2-fixing culture (Fig. S2 A,B), consistent with the results of direct P_{700} determinations (Fig. 5) as well as the concomitantly increasing PSII abundance. Changes in phycobilisome fluorescence normalized to PSII was statistically insignificant during the dark/light cycle in both types of cultures, however, it was slightly higher in the N_2-fixing culture (Fig. S2 C,D).

Overall, data indicate that the absolute phycobiliprotein content (including both allophycocyanin and phycocyanin) is lower in diazotrophic cultures compared to NO_3^- cultures (Tables 1, 2). Note that the low temperature fluorescence emission spectra, normalized to the 695 nm Chl a emission peak, do not reflect this lower phycobilisome content of the N_2-fixing cultures (rather, it shows an increase) because it is expressed relatively to the cellular Chl a content, which was also lower in N_2-fixing cultures (Fig. S2, Tables 1, 2).

Population dynamics

The optical density (OD) recorded in real time by the photobioreactor sensors shows that population dynamics are highly reproducible across repeated days within a bioreactor (data not shown) and across independent culture replicates. A typical record covering a 24h light cycle is shown in Fig. 1. OD_{720} is a measure of light scattering due to particulate material, and increases as suspended cells grow in number and/or size, or accumulate intracellular material such as storage granules (Polerecky et al., *in press*), while OD_{680} or (OD_{680} - OD_{720}) mainly accounts for Chl *a* absorption. The relationship between OD and cell abundance is not linear, and an equal OD of two cultures may not reflect the same cell concentration, due to various factors impacting the optical signal (Table 1). Nevertheless, OD is a sensitive proxy for changes in biomass dynamics and, in this respect, can be used to infer growth dynamics. Hence, we used the rates of change in OD_{720} between dilution events to determine transient growth or biomass accumulation in each culture (Fig. S3).

Oxygen and pH are two key proxies of biological processes. Super- or sub-saturating O_2 levels, i.e above or below the predicted physical equilibrium (dotted lines in Fig. 2A and B) reflect photosynthetic O_2 evolution, and/or respiration. In the absence of any buffer, the fluctuations in pH would directly be related to the consumption (or release) of CO_2 in the cultures. In that case, a slope difference, e.g. in the initial pH rise would clearly indicate a more intense DIC consumption in the N_2 fixing culture. However, the presence of a buffering agent in the culture medium partially counteracts the pH rise as DIC is consumed, and variations in pH are therefore not only related to biological but also to physicochemical buffering effects. In the present experiment, TAPS was used with a p*Ka* = 8.3, thus, pH variations were actually more strongly constrained in the NO_3^- culture, whose pH values are closer to that p*Ka*, where buffering capacity is the highest, which then may partly explain the pH slope difference between the NO_3^- and the N_2 fixing cultures.

In both treatments, a rapid O_2 increase within the first 45 min of light phase indicates the initiation of photosynthetic activity; it is about 1.5-fold faster in the NO_3^- cultures (99.1 ±1.7 µmol O_2 L^{-1} h^{-1}) as compared to the N_2-fixing cultures 67.8 ±1.2 µmol O_2 L^{-1} h^{-1}). The OD_{720} concomitantly rises as a consequence of photosynthesis-mediated carbon incorporation, but the initial OD_{720} signal at the dark to light transition is significantly lower in the N_2-fixing cultures compared to the NO_3^- cultures. This difference is likely due to night-time respiration of intracellular carbon reserves in N_2-fixing cells that further deplete carbohydrate reserves compared to the NO_3^- cultures. The first dilution event at the beginning of each light phase is therefore delayed 1.25 h in the N_2 in comparison to the NO_3^- culture (Fig 1 black, down arrows). The photosynthesis-mediated carbon uptake draws down DIC in the cultures: after 2 h of the light cycle, DIC in N_2-fixing cultures is

only 87 ±59 µmol L^{-1} whereas it is 311 ±47 µmol L^{-1} in NO_3^- cultures. This is also reflected in the changes in pH; initially constant (7.84 ±0.02 in the NO_3^- culture vs. 7.58 ±0.04 in the N_2-fixing cultures) for about 0.5 h at the beginning of the light period, the pH then increases continuously during the next 3 (in the NO_3^- cultures) or 2 h (in the N_2-fixing cultures). The fast decline in O_2 concentrations which follows (-49 µmol O_2 L^{-1} h^{-1} in the NO_3^- culture and -90 µmol O_2 L^{-1} h^{-1} in the N_2 fixing culture) reflects the onset of DIC limitation.

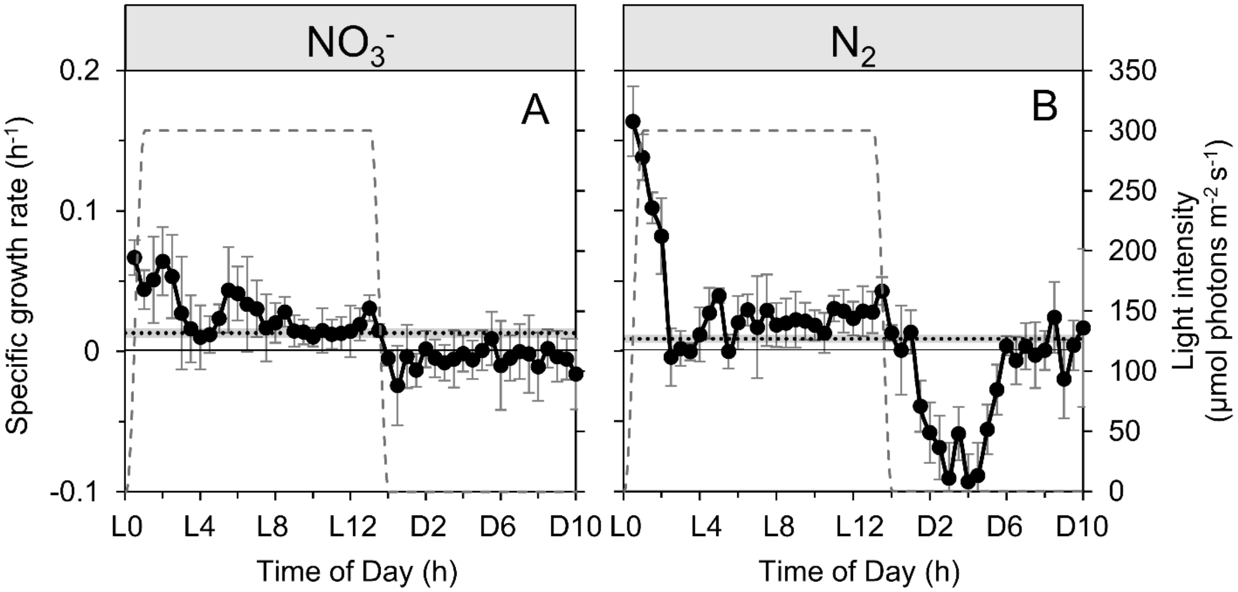

Figure S3. Dynamics of instantaneous, specific growth rates of *Cyanothece* grown in ASP 2 medium supplemented with NO_3^- (A) or under obligate diazotrophy (B), as estimated from the changes in the OD_{720} signal recorded in the photobioreactor. The dotted lines show the average specific growth rate over the 24h period, with their respective standard deviations (grey shaded areas). The data points of specific growth rate are averages of 3 - 10 individual dilution steps within 2 (N_2-fixing cultures) or 3 (NO_3^- cultures) independent photobioreactors. Error bars represent standard deviations. The grey dashed line represents the light profile throughout the day.

The extent of DIC limitation was assessed by running an additional culture in which the air bubbling was enriched with CO_2. A Gas Mixing System (GMS 150, Photon System Instruments Ltd., Brno, CZ) that precisely mixes air (sourced by a compressor) and pure CO_2 (Linde Gas, 99.5% purity), delivered a CO_2-air mixture with a final concentration of 2000 ppm CO_2. The release of DIC limitation was observed by monitoring the temporal dynamics of oxygen concentration in the culture (Fig. S4). CO_2 enrichment in the bubbling immediately releases light-phase DIC limitation in the culture (compare with Fig. 2 A,B) and photosynthesis then operates more efficiently, as shown by a higher oxygen release throughout the light phase.

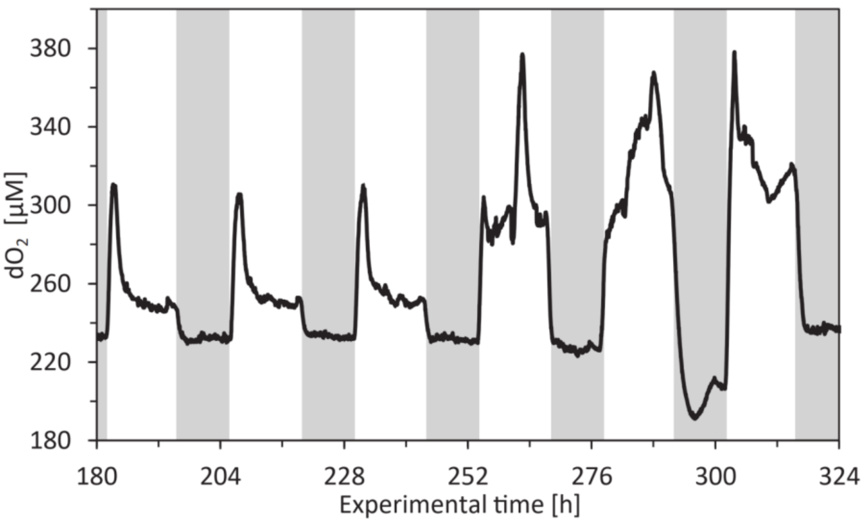

Figure S4 Monitoring of oxygen concentration in an NO_3^- culture, when CO_2-enriched air bubbling was activated at hour 254 (beginning of the light phase). Bubbling with CO_2 unleashes an increase in O_2 and big drop in O_2 at night implying a quick increase of respiration before N fixation. Note that O_2 probe calibration here in Figure S4 is offset from the correct calibration of the O_2 values presented in Figure 2.

Gross and net O_2 fluxes were measured by Membrane Inlet Mass Spectrometry (MIMS; Fig. S5) following the $^{18}O_2$-based approach described by Fock & Sültemeyer (1998). The measurements were performed with a Prisma 200 quadrupole mass spectrometer (Pfeiffer Vacuum, Asslar, Germany) connected to a custom-made, temperature-controlled cuvette (5 ml volume) via a stainless steel sampling finger covered by a thin silicone membrane stretched over its flat perforated head. Prior to measurements, cultures were concentrated by gentle centrifugation and subsequently dissolved in fresh ASP 2 medium without NO_3^-. $^{18}O_2$ gas was injected in the vial and incubated for ca. 30 min with repeated shaking to aid gas dissolution prior to transfer of the sample to the MIMS cuvette. The production of $^{16}O_2$ and uptake of $^{16}O_2$ and $^{18}O_2$ were then monitored simultaneously in consecutive dark and light (300 µmol photons m^{-2} s^{-1}) phases lasting 3 min each. The O_2 signals were calibrated with air-bubbled and N_2-bubbled medium (or addition of Na-dithionite, which yielded comparable results for the 0% O_2 signal as N_2 bubbling). Signals were corrected for abiotic O_2 consumption/leakage during the measurements by subtracting slopes of $^{18}O_2$ and $^{16}O_2$ determined in fresh media (spiked with $^{18}O_2$ or air-bubbled, respectively), and for any non-biological fluctuations using the argon signal recorded simultaneously during each measurement. These measurements were performed between 9 and 11 am.

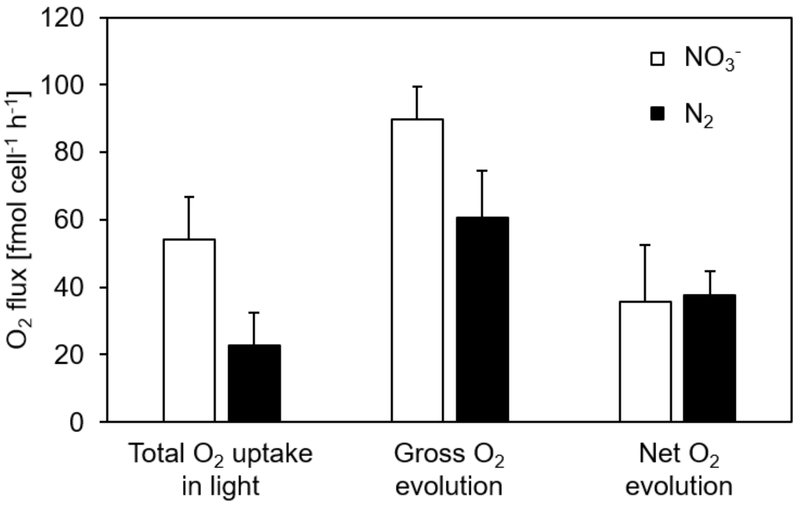

Figure S5 O_2 uptake and production by *Cyanothece* grown with (NO_3^-) or without NO_3^- (N_2) as determined between L2 and L4 by Membrane Inlet Mass Spectrometry. An 18O2-based approach was used to differentiate between simultaneous production and uptake of O2 in the light. Total O_2 uptake in the light reflects the sum of dark respiration and light-dependent O_2 uptake mediated by Mehler reaction, flv-dependent O_2 uptake and/or photorespiration (i.e., oxygenase function of RubisCO). Gross O_2evolution is calculated as the sum of net O_2 evolution and total O_2 uptake in the light. Error bars show stdev with n = 3.

In a second approach, MIMS was also used to monitor CO_2 draw-down in the cultures. This measurement was performed about 2-3 hours before the end of the light phase (Fig. S6). The uptake and release of CO_2 as well as the production of $^{16}O_2$ and the uptake of $^{16}O_2$ and $^{18}O_2$ by the culture were followed simultaneously over time. The CO_2 signal was calibrated with NaOH (0% CO_2) and by adding defined amounts of $NaHCO_3$ to 0.1 M HCl (where all DIC is present in the form of CO_2).

In the light, initial CO_2 concentrations in the cuvette gradually decreased due to cellular carbon uptake (mass 44, Fig. S4), while O_2 evolution due to photosynthetic water splitting was reflected in an increase in $^{16}O_2$ concentration (mass 32, Fig. S4) and the concurrent uptake of O_2 was reflected in a decrease in the concentration of the isotopically labeled $^{18}O_2$ (mass 36, Fig. S6). After this initial phase of net photosynthesis and associated CO_2 draw-down, CO_2 uptake as well as net O_2 evolution stopped abruptly, while the rate of O_2 uptake strongly increased (see black arrow, Fig. S6). We hypothesize that at this time point, RubisCO started to function as an oxidase as the CCM was not able to supply enough CO_2 to support the carboxylation reaction. Accordingly, the minimum CO_2 concentration of 0.5 umol L^{-1} reached at this point represents an affinity limit of the *Cyanothece* CCM. Notably, this concentration is equivalent to the CO_2 concentration predicted at the minimum DIC level we observed independently in the bioreactors, suggesting that DIC was consumed down to the affinity limit of the *Cyanothece* CCM in both N treatments.

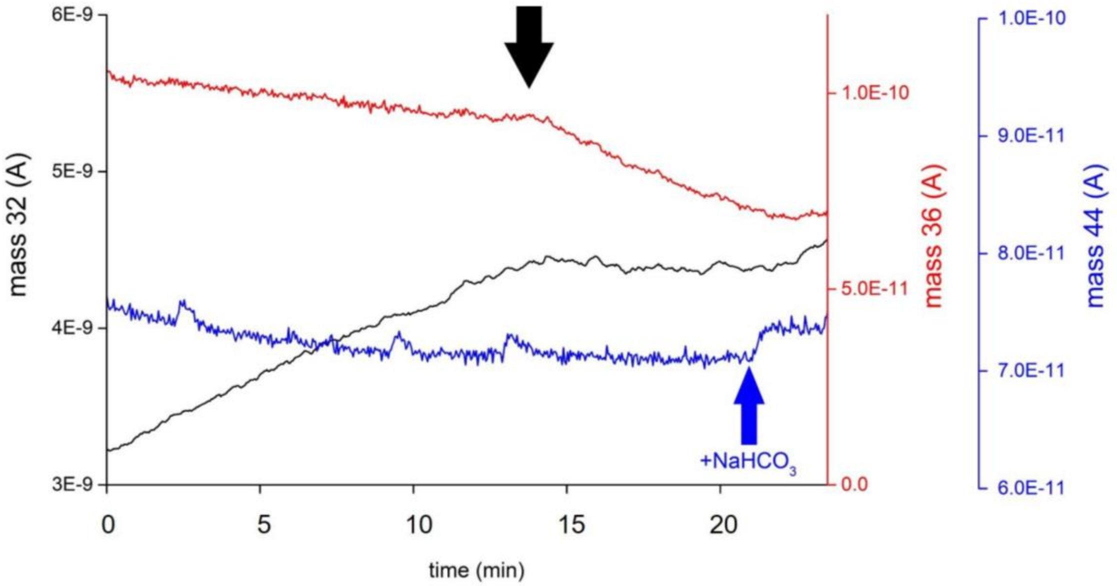

Figure S6 Changes in abundance (A) of masses 32 ($^{16}O_2$), 36 ($^{18}O_2$) and 44 (CO_2) measured simultaneously by Membrane Inlet Mass Spectrometry (MIMS) on a culture of *Cyanothece* maintained in obligate diazotrophy. Decrease in mass 44 reflects cellular carbon uptake, increase in mass 32 reflects net evolution of $^{16}O_2$ by photosynthetic water splitting, decrease in mass 36 reflects uptake of $^{18}O_2$ by dark respiration, photorespiration and Mehler reaction. Note the ceasing of CO_2 uptake as well as net evolution of $^{16}O_2$ and the concomitant acceleration of $^{18}O_2$ uptake, which reflects the induction of photorespiration by RubisCO, at the time point highlighted by the black arrow. The CO_2 concentration at this time point presumably reflects the affinity limit of the CCM of *Cyanothece*. Note that the responses in O_2 fluxes to CO_2 limitation could be relieved by addition of 100 umol L^{-1} $NaHCO_3$ (blue arrow). A similar response to CO_2 draw-down was observed in two replicate measurements (data not shown).

References

Bennett, A., and Bogorad, L. (1973) Complementary chromatic adaptation in a filamentous blue-green alga. J Cell Biol, 58, 419-435.

Dean, A.P., Nicholson, J.M. and Sigee, D.C. (2008) Impact of phosphorus quota and growth phase on carbon allocation in *Chlamydomonas reinhardtii*: an FTIR microspectroscopy study. Eur J Phycol, 43, 345-354.

Fock HP and Sültemeyer DF (1989): O_2 evolution and uptake measurements in plant cells by mass spectrometer. In: Modern Methods of Plant Analysis Vol 9. HF Liskens and JF Jackson, Springer-Verlag, Heidelberg, Germany: 3-18.

Gantt, E. (1980) Structure and function of phycobilisomes: light harvesting pigment complexes in red and blue-green algae, Int Rev Cytol 66: 45-80

Grossman, A.R., Bhaya, D., & He, Q. (2001) Tracking the light environment by cyanobacteria and the dynamic nature of light harvesting. J Biol Chem 276(15):11449-52

Hall P. O. J. and Aller R. C. (1992) Rapid, small-volume, flow injection analysis for CO_2 and NH_4 in marine and freshwaters. Limnol Oceanogr 37, 1113–1119.

Messineo, L. (1966) Modification of the Sakaguchi reaction: Spectrophotometric determination of arginine in proteins without previous hydrolysis. Arch Biochem Biophys 117, 534-540.

Polerecky L, Masuda T, Eichner M, Rabouille S, Vancová M, Kienhuis MVM, Bernát G, Bonomi-Barufi J, Campbell DA, Claquin P, Červený J, Giordano M, Kotabová E, Kromkamp J, Lombardi AT, Lukeš M, Prášil O, Stephan S, Suggett D, Zavřel T and Halsey KH (2021) Temporal patterns and intra- and inter-cellular variability in carbon and nitrogen assimilation by the unicellular cyanobacterium *Cyanothece* sp. ATCC 51142. Front Microbiol 12:620915. doi: 10.3389/fmicb.2021.620915.

Zavřel, T., Sinetova, M.A., and Červený, J. (2015). Measurement of chlorophyll a and carotenoids concentration in cyanobacteria. Bio-protocol 5, e1467.

Zavřel, T., Očenášová, P., Sinetova, M.A., and Červený, J. (2018). Determination of storage (starch/glycogen) and total saccharides content in algae and cyanobacteria by a phenol-sulfuric acid method. Bio-protocol 8, e2966.
